# Efficacy and safety of GV1001 in patients with moderate-to-severe Alzheimer’s disease already receiving donepezil: a phase 2 randomized, double-blind, placebo-controlled, multicenter clinical trial

**DOI:** 10.1186/s13195-021-00803-w

**Published:** 2021-03-26

**Authors:** Seong-Ho Koh, Hyuk Sung Kwon, Seong Hye Choi, Jee Hyang Jeong, Hae Ri Na, Chan Nyoung Lee, YoungSoon Yang, Ae Young Lee, Jae-Hong Lee, Kyung Won Park, Hyun Jeong Han, Byeong C. Kim, Jin Se Park, Jee-Young Lee, Sangjae Kim, Kyu-Yong Lee

**Affiliations:** 1 Department of Neurology, Hanyang University Guri Hospital, Hanyang University College of Medicine, 153, Gyeongchun-ro, Guri, 11923 South Korea; 2 Department of Translational Medicine, Hanyang University Graduate School of Biomedical Science & Engineering, Seoul, 04763 South Korea; 3 Department of Neurology, Inha University School of Medicine, Incheon, 22332 South Korea; 4 Department of Neurology, Ewha Womans University School of Medicine, Seoul, 07985 South Korea; 5 Department of Neurology, Bobath Memorial Hospital, Seongnam, 13552 South Korea; 6 Department of Neurology, Korea University Anam Hospital, Seoul, 02856 South Korea; 7 Department of Neurology, Veterans Health Service Medical Center, Seoul, 05368 South Korea; 8 Department of Neurology, Chungnam National University Hospital, Daejeon, 35015 South Korea; 9 Department of Neurology, Asan Medical Center, Seoul, 05505 South Korea; 10 Department of Neurology, Dong-A University Hospital, Busan, 49201 South Korea; 11 Department of Neurology, Myongji Hospital, Hanyang University College of Medicine, Goyang, 10475 South Korea; 12 Department of Neurology, Chonnam National University Hospital, Gwangju, 61469 South Korea; 13 Department of Neurology, Inje University Haeundae Paik Hospital, Buasn, 48108 South Korea; 14 Department of Neurology, Seoul National University Boramae Medical Center, Seoul, 07061 South Korea; 15 Teloid Inc., 920 Westholme Ave, Los Angeles, CA 90024 USA

**Keywords:** GV1001, Efficacy, Safety, Alzheimer’s disease, Clinical trial

## Abstract

**Background:**

Our previous studies showed that GV1001 has various protective effects against β-amyloid and other stressors. Based on these findings, we hypothesized that GV1001 might have beneficial effects in patients with Alzheimer’s disease (AD).

**Methods:**

A phase 2, double-blind, parallel-group, placebo-controlled, 6-month randomized clinical trial was performed to evaluate the safety and efficacy of subcutaneously administered GV1001. Between September 2017 and September 2019, 13 centers in South Korea recruited participants. A total of 106 patients were screened, and 96 patients with moderate-to-severe AD were randomized 1:1:1 to the placebo (group 1, *n* = 31), GV1001 0.56 mg (group 2, *n* = 33), and 1.12 mg (group 3, *n* = 32) groups. GV1001 was administered every week for 4 weeks (4 times), followed by every 2 weeks until week 24 (10 times). The primary endpoint was the change in the Severe Impairment Battery (SIB) score from baseline to week 24. The key secondary efficacy endpoints were the change in the Clinical Dementia Rating Sum of Box (CDR-SOB), Alzheimer’s Disease Cooperative Study-Activities of Daily Living (ADCS-ADL), Neuropsychiatric Inventory (NPI), Mini-Mental State Examination, and Global Deterioration Scale scores. The safety endpoints were also assessed based on adverse events, laboratory test results, vital signs, and other observations related to safety.

**Results:**

Group 3 showed less decrease in the SIB score at 12 and 24 weeks compared with group 1 (*P* < 0.05). These were not significantly observed in group 2. Among the secondary endpoints, only the NPI score showed significantly better improvement in group 2 than in group 3 at week 12; however, there were no other significant differences between the groups. Although the ADCS-ADL and CDR-SOB scores showed a pattern similar to SIB scores, a statistically significant result was not found. Adverse events were similar across all three groups.

**Conclusions:**

The results indicate that GV1001 1.12 mg met the primary endpoint of a statistically significant difference. GV1001 was well tolerated without safety concerns. This study warrants a larger clinical trial.

**Trial registration:**

ClinicalTrials.gov NCT03184467. Registered on June 12, 2017.

**Supplementary Information:**

The online version contains supplementary material available at 10.1186/s13195-021-00803-w.

## Background

As the global population is aging, the number of patients with Alzheimer’s disease (AD) has increased and is now a global healthcare challenge [1, 2]. AD, the most common neurodegenerative disease that causes progressive dementia, is characterized by a loss of neurons and synapses in the cerebral cortex, mainly in the temporal and parietal lobes. The most important pathological findings of AD include senile plaques caused by β-amyloid (Aβ) and neurofibrillary tangles due to hyperphosphorylated tau [3]. Neuroinflammation is another important pathological finding that accompanies AD [4]. A total of five drugs have been approved for the symptomatic treatment of AD, from tacrine in 1993 to memantine in 2003. However, they did not modify the pathologic progression, and tacrine treatment was discontinued in 2013 because of its hepatotoxicity. Currently, several late-stage development programs for disease-modifying drugs, predominantly targeting not only amyloid but also tau and neuroprotection, are in the pipeline [5, 6]. Among the anti-amyloid agents, aducanumab has been applied for US Food and Drug Administration (FDA) marketing approval, based on two phase 3 clinical trials (EMERGE and ENGAGE trial) with conflicting results [6, 7]. Nevertheless, there are huge unmet medical needs for AD worldwide.

Except for the above-described drugs, no drug has been approved for AD despite countless drug development efforts [8–10]. Several experts agree that the majority of newly developed drugs in clinical trials have failed because they targeted a single aspect of AD [9, 11–13]. Potential therapeutics should have multiple effects, including a reduction in senile plaques and neurofibrillary tangles, instead of a single effect.

GV1001 is a peptide consisting of 16 amino acids that correspond to a fragment from the catalytic site of human telomerase reverse transcriptase [14]. We have previously confirmed that GV1001 protects neural cells against neurotoxicity, apoptosis, and reactive oxygen species (ROS) induced by Aβ and oxidative stress [14–16]. These neuroprotective effects are mediated through anti-apoptotic, mitochondrial stabilizing, anti-inflammatory, anti-aging, and anti-oxidant effects [14–16]. These findings suggest that GV1001 has diverse modes of action against AD; thus, we hypothesized that these protective effects would make GV1001 a promising therapeutic option for AD. Therefore, this study aimed to assess the effect of GV1001 on the cognition and activities of daily living in patients with moderate-to-severe AD.

## Methods

### Study design and participants

This multicenter, randomized, double-blind, placebo-controlled, parallel design, and prospective phase 2 clinical trial was performed in neurology departments in 13 hospitals throughout South Korea (five hospitals in Seoul, two hospitals in Busan, and six hospitals across Guri, Incheon, Seongnam, Daejeon, Goyang, and Gwangju). Participants aged 55–85 years; those who were clinically diagnosed with probable AD, defined by the National Institute of Neurological and Communicative Disorders and Stroke-Alzheimer’s Disease and Related Disorders Association criteria and the Diagnostic and Statistical Manual of Mental Disorders, 4th edition, criteria [17, 18]; those who had a Korean Mini-Mental State Examination (K-MMSE) score ≤ 19 at the screening visit; those who had a Global Deterioration Scale (GDS) score of 5–6; and those who were receiving stable doses of donepezil (10 mg) for > 3 months before the screening visit were included in the study (Additional file 2, Table S1). All patients underwent medical and neurologic evaluations, including magnetic resonance imaging (MRI) and computed tomography (CT), and the clinical diagnosis was made by investigators at each site. Patients with a GDS score of 7 were not assessed for eligibility, as they were unable to perform cognitive tests and follow the clinical trial protocol owing to loss of linguistic ability and fundamental motor ability, such as walking.

Table S1 in Additional file 2 shows the detailed exclusion criteria. Briefly, patients with any other cause of dementia based on MRI/CT findings and neurological examination within 12 months of randomization, those with current clinically significant psychiatric conditions or history of such conditions, those with a history of known or suspected seizures, or those using drugs other than donepezil in the treatment of AD were excluded from the study.

The detailed protocol and its amendments (Additional file 1) were performed according to the Good Clinical Practice guidelines and the Declaration of Helsinki and approved by the independent institutional review boards of each participating center. The protocol was registered on ClinicalTrials.gov (NCT03184467).

### Randomization, masking, and procedure

The study consisted of a screening visit (up to 2 weeks before the first dose of GV1001), 24-week double-blind treatment period, and end-of-study visit. The eligible patients were enrolled by the investigators at each center and randomly assigned in a 1:1:1 ratio using the Interactive Web Response System to the GV1001 0.56 mg, GV1001 1.12 mg, or placebo (normal saline) group. The study treatment (GV1001 0.56 mg, GV1001 1.12 mg, or placebo) was administered by subcutaneous (SC) injection every week for 4 weeks (4 times), followed by SC administration every 2 weeks until week 24 (10 times) for a total of 14 SC injections. The placebo was visually identical to the investigational product. All investigators, participants, and care providers were blinded to the treatment assignment throughout the study. The unblinding can be conducted by performing database locking, and the information can be confirmed only with the approval of the steering committee. Efficacy evaluations were performed at baseline, week 12, and week 24. Safety was also assessed throughout the study. All investigators, patients, and care providers were blinded to the treatment assignment during the study.

### Outcome

We evaluated the safety and efficacy of GV1001 (0.56 mg or 1.12 mg) administered subcutaneously as a treatment for moderate-to-severe AD. The primary efficacy endpoint was the change in the Severe Impairment Battery (SIB) score from baseline to week 24 [19]. SIB was filled out by the same person, a well-educated and trained expert, during the follow-up, and most patients were living at home. The secondary endpoints included the effects of GV1001 on the Clinical Interview-Based Impression of Change, Clinician Interview-Based Impression of Change plus caregiver input (CIBIC-plus), Clinical Dementia Rating Sum of Box (CDR-SOB) [20], Alzheimer’s Disease Cooperative Study-Activities of Daily Living (ADCS-ADL) [21], Neuropsychiatric Inventory (NPI) [22], MMSE [23], and GDS scores [24]. These secondary endpoints were also assessed based on the change from baseline to week 24. The safety endpoints were assessed based on adverse events (AEs), laboratory test results, vital signs, and other observations related to safety (including electrocardiogram findings and physical examination).

### Statistical analysis

The sample size for this study to evaluate the efficacy was not determined based on statistical considerations because GV1001 had a different mechanism of action and effectiveness from other existing medications. However, the sample size was determined by examining and comparing the domestic and overseas literature reviews of multicenter, randomized, double-blind, placebo-controlled, parallel design, and prospective phase 2 clinical studies. We determined that a sample size of 78 (26 for each group) was needed to compare the difference in changes of SIB scores from baseline to week 24 in the three groups. Estimating a 20% dropout rate, we planned to enroll a total of 96 patients. According to the prespecified protocol (Additional file 1), the efficacy endpoints (change from baseline to week 24 in the SIB, CDR-SOB, ADCS-ADL, NPI, MMSE, and GDS scores) were mainly assessed using the full analysis set (FAS), which included all randomly assigned participants who received at least one dose of study medication and underwent at least one assessment of the primary endpoints, although some participants did not complete the protocol. Additionally, we analyzed the per-protocol set (PPS), which only included the participants who completed the protocol. The safety endpoints were assessed with the safety set, which included all randomly assigned participants who received at least one dose of study medication.

The chi-square test and analysis of variance were used for baseline demographics. The differences between the treatment and placebo groups were assessed using a mixed effects model repeated measures (MMRM) analysis [25]. The comparison for MMRM was based on the model with primary and secondary efficacy scores as dependent variables, independent variables of the treatment group (group 3 [GV1001 1.12 mg] and placebo, or group 2 [GV1001 0.56 mg] and placebo), post-baseline visits (visit 9 and visit 15), treatment by week interaction as fixed effects, and baseline score as covariate. The covariance structure was set as unstructured. The change from baseline in the CIBIC-plus score to week 24 was analyzed using Pearson’s chi-square test. Comparisons between groups were performed using a two-sided significance level of 0.05. All statistical analyses were performed using SAS version 9.4. This trial is registered with ClinicalTrials.gov (NCT03184467).

## Results

**Fig. 1:**
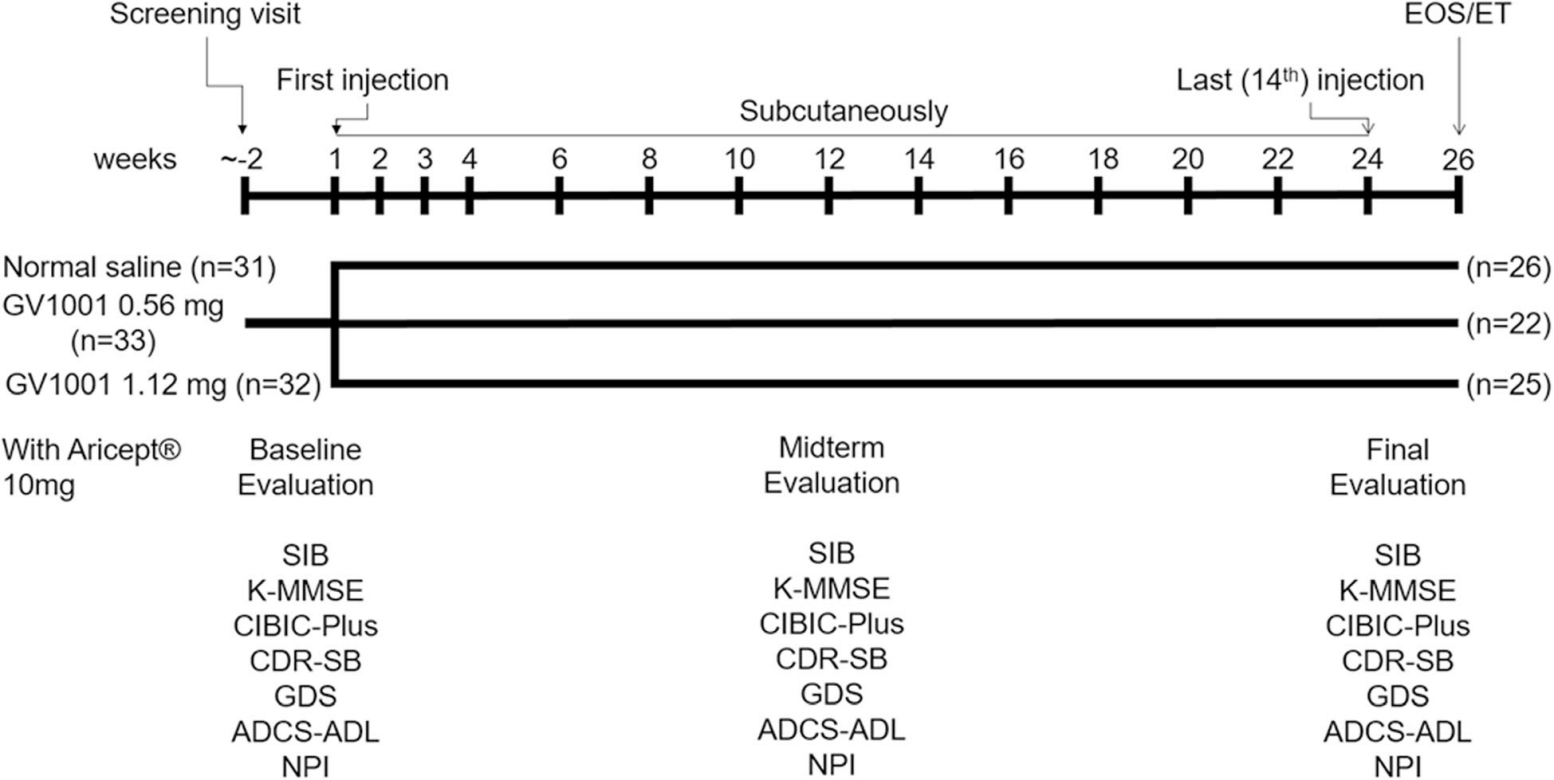
Study design. Overall study design of the present clinical trial

**Fig. 2:**
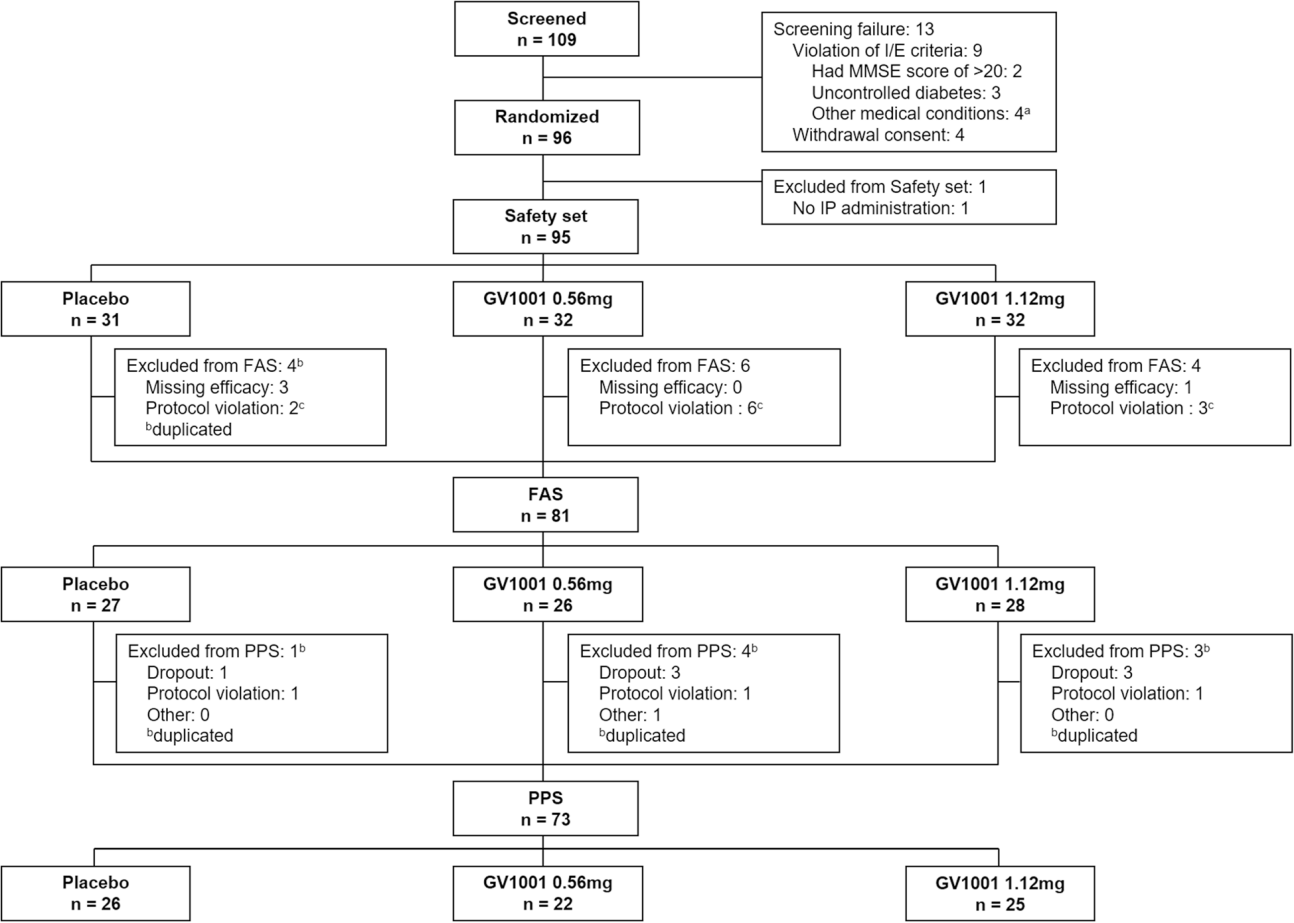
Patient disposition. Participant flow throughout the trial. ^a^Other medical conditions include renal dysfunction (*n* = 1), hepatic dysfunction (*n* = 2), and condition that in the opinion of the investigator can interfere with the interpretation of the study result (*n* = 1). ^b^One participant was excluded according to multiple reasons. ^c^Participants from a single center were excluded after the administration of the first study treatment, as mandatory clinical data, including baseline clinical data and the results of neurological examination performed at corresponding time points, were not uploaded to the central web-based system and the investigator of this center could not verify the source of these data. Abbreviations: *MMSE* Mini-Mental State Examination, *I/E* inclusion/exclusion, *IP* investigational product, *FAS* full analysis set, *PPS* per-protocol set

Between September 2017 and September 2019, a total of 109 participants were assessed for eligibility; subsequently, 96 participants were enrolled and randomized into three groups: group 1 (placebo; *n* = 31), group 2 (GV1001 0.56 mg; *n* = 32), and group 3 (GV1001 1.12 mg; *n* = 32) (Fig. 1). Of the 96 participants, 26 participants in group 1, 22 participants in group 2, and 25 participants in group 3 completed the 6-month follow-up (Fig. 1). The safety set included 31 participants in group 1, 32 participants in group 2, and 32 participants in group 3. The FAS included 27 participants in group 1, 26 participants in group 2, and 28 participants in group 3. The PPS included 26 participants in group 1, 22 participants in group 2, and 25 participants in group 3 (Fig. 2).

**Fig. 3:**
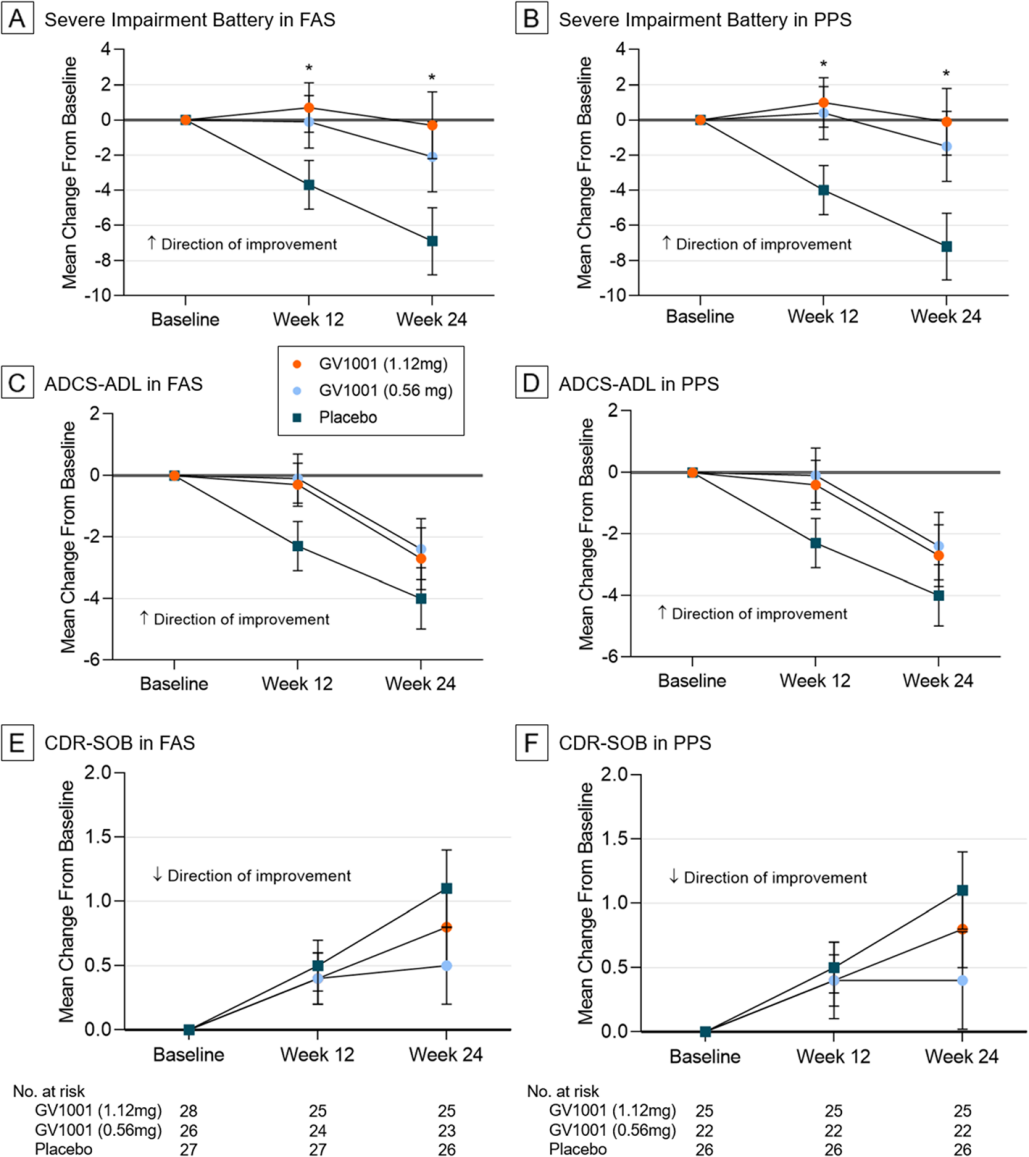
Effects of GV1001 on SIB, ADCS-ADL, and CDR-SOB scores in patients with AD with moderate-to-severe dementia. The change in the SIB score from baseline to 24 weeks was considered the primary endpoint. In the FAS and PPS, the patients assigned 1.12 mg of GV1001 had significantly better mean change from the baseline scores than the placebo group at weeks 12 and 24 (**a** and **b**, respectively). Among the secondary endpoints, ADCS-ADL (**c**, **d**) and CDR-SOB (**e**, **f**) scores showed a similar pattern to the SIB scores; however, statistical significance was not achieved in the FAS and PPS. LS indicates least squares. Error bars indicate standard error. *P < 0.05 (group 2 vs. group 3). Abbreviations: *SIB* Severe Impairment Battery, *ADCS-ADL* Alzheimer’s Disease Cooperative Study-Activities of Daily Living, *CDR-SOB* Clinical Dementia Rating Scale-Sum of Boxes, *AD* Alzheimer’s disease, *FAS* full analysis set, *PPS* per-protocol set, *LS* least square

Significant differences in baseline characteristics, including age, sex, and age at AD diagnosis, years since diagnosis, and K-MMSE, CDR-SOB, NPI, GDS, ADCS-ADL, and Clinician Interview-based Impression of Severity scores were not observed between the three groups (Table 1 and Additional file 2, Table S3). As a primary endpoint, the change in the SIB score from baseline to week 24 was evaluated in the FAS and PPS. At week 24, the difference between group 1 (normal saline) and group 3 (GV1001 1.12 mg) was 6.6 in the FAS (*p* = 0.027) and 7.1 in the PPS (*p* = 0.018) (Table 2 and Fig. 3a, b, respectively). This statistically significant difference between group 1 and group 3 was also observed at week 12 in both FAS (*p* = 0.030) and PPS (*p* = 0.016) (Table 2 and Fig. 3a, b). However, the difference between group 1 (normal saline) and group 2 (GV1001 0.56 mg) was not statistically significant in the FAS and PPS, although it showed a slight tendency in favor of group 2. Among the secondary endpoints, only NPI showed significantly better improvement in group 3 than in group 1 at week 12 (Table 2); however, there were no other significant differences between the groups. Although the ADCS-ADL and CDR-SOB scores showed a pattern similar to SIB scores, a statistically significant result was not observed (Fig. 3c–f and Tables S3–6 in Additional file 2).

**Table 1:** Demographic and baseline characteristics

	Placebo (group 1)	GV1001 0.56 mg (group 2)	GV1001 1.12 mg (group 3)	Overall	*P* value
	*n* = 31	*n* = 33	*n* = 32	*n* = 96	
Age (years)					0.821^a^
Mean ± SD	70.9 ± 8.9	70.2 ± 9.8	71.6 ± 8.4	70.9 ± 9.0	
Median	72.0	73.0	75.0	74.0	
Min, Max	56.0, 84.0	56.0, 85.0	56.0, 82.0	56.0, 85.0	
Sex					0.390^b^
Male, *n* (%)	12 (38.7)	10 (30.3)	15 (46.9)	37 (38.5)	
Female, *n* (%)	19 (61.3)	23 (69.7)	17 (53.1)	59 (61.5)	
Age at AD diagnosis (years)					0.671^a^
Mean ± SD	67.9 ± 8.6	66.2 ± 8.3	67.8 ± 8.4	67.3 ± 8.4	
Median	68.0	66.0	70.0	68.0	
Min, Max	54.0, 82.0	53.0, 83.0	50.0, 80.0	50.0, 83.0	
Years since diagnosis of AD					0.415^a^
Mean ± SD	3.6 ± 1.7	4.6 ± 3.9	4.4 ± 2.6	4.2 ± 2.9	
Median	3.0	3.0	4.0	3.5	
Min, Max	1.0, 8.0	0.0, 14.0	1.0, 10.0	0.0, 14.0	
SIB					0.959^a^
Mean ± SD	75.9 ± 16.5	77.3 ± 19.7	76.9 ± 20.1	76.7 ± 18.7	
Median	81.0	85.0	84.0	83.0	
Min, Max	39.0, 97.0	21.0, 97.0	15.0, 96.0	15.0, 97.0	
K-MMSE					0.618^a^
Mean ± SD	11.9 ± 4.9	13.1 ± 5.1	12.1 ± 5.3	12.4 ± 5.1	
Median	12.0	14.0	13.0	13.0	
Min, Max	4.0, 19.0	2.0, 19.0	1.0, 19.0	1.0, 19.0	
CDR-SOB					0.773^a^
Mean ± SD	10.1 ± 4.1	9.5 ± 4.4	10.1 ± 4.1	9.9 ± 4.2	
Median	10.0	9.5	10.0	10.0	
Min, Max	4.0, 18.0	3.0, 22.0	5.0, 23.0	3.0, 23.0	
NPI					0.077^a^
Mean ± SD	18.8 ± 14.4	12.5 ± 12.0	22.6 ± 16.7	17.9 ± 15.0	
Median	14.0	6.5	18.0	14.0	
Min, Max	1.0, 63.0	1.0, 50.0	4.0, 68.0	1.0, 68.0	
GDS					0.294^a^
Mean ± SD	5.4 ± 0.5	5.2 ± 0.4	5.3 ± 0.5	5.3 ± 0.5	
Median	5.0	5.0	5.0	5.0	
Min, Max	5.0, 6.0	5.0, 6.0	5.0, 6.0	5.0, 6.0	
ADCS-ADL					0.141^a^
Mean ± SD	32.9 ± 9.9	38.0 ± 10.6	35.8 ± 10.3	35.6 ± 10.4	
Median	33.0	41.5	35.5	35.0	
Min, Max	12.0, 54.0	10.0, 52.0	14.0, 51.0	10.0, 54.0	
CIBIS					0.741^a^
Mean ± SD	4.7 ± 0.9	4.7 ± 0.7	4.8 ± 0.8	4.7 ± 0.8	
Median	5.0	5.0	5.0	5.0	
Min, Max	3.0, 6.0	3.0, 6.0	3.0, 6.0	3.0, 6.0	

*Abbreviations*: *AD* Alzheimer’s disease, *SD* standard deviation, *SIB* Severe Impairment Battery, *K-MMSE* Korean Mini-Mental State Examination, *CDR-SOB* Clinical Dementia Rating Scale-Sum of Boxes, *NPI* Neuropsychiatric Inventory, *GDS* Global Deterioration Scale, *ADCS-ADL* Alzheimer’s Disease Cooperative Study-Activities of Daily Living, *CIBIS* Clinician Interview-Based Impression of Severity

^a^Analysis of variance and ^b^chi-square test were used

**Table 2:** Effects of GV1001 on primary and secondary endpoints in FAS and PPS

Variables	Week	LS mean of CFB (± SE) in FAS				
		Placebo (group 1)	GV1001 0.56 mg (group 2)	*P* value	GV1001 1.12 mg (group 3)	*P* value
		*n* = 27	*n* = 26		*n* = 28	
SIB	12	− 3.7 (1.4)	− 0.1 (1.5)	0.178	0.7 (1.4)*	0.030
	24	− 6.9 (1.9)	− 2.1 (2.0)	0.097	− 0.3 (1.9)*	0.027
K-MMSE	12	− 0.0 (0.5)	− 0.8 (0.6)	0.215	0.2 (0.5)	0.858
	24	− 0.7 (0.6)	− 1.2 (0.6)	0.365	− 0.4 (0.6)	0.751
CDR-SOB	12	0.5 (0.2)	0.4 (0.2)	0.772	0.4 (0.2)	0.594
	24	1.1 (0.3)	0.5 (0.3)	0.182	0.8 (0.3)	0.518
NPI	12	4.6 (2.6)	0.9 (2.6)	0.263	− 4.8 (2.4)*	0.018
	24	− 1.6 (4.3)	3.5 (4.3)	0.419	− 2.1 (4.3)	0.792
GDS	12	− 0.1 (0.1)	0.0 (0.1)	0.203	− 0.1 (0.1)	0.664
	24	− 0.1 (0.1)	0.0 (0.1)	0.075	− 0.1 (0.1)	0.476
ADCS-ADL	12	− 2.3 (0.8)	− 0.1 (0.8)	0.148	− 0.3 (0.7)	0.166
	24	− 4.0 (1.0)	− 2.4 (1.0)	0.451	− 2.7 (1.0)	0.420
Variables	Week	LS mean of CFB (± SE) in PPS				
		Placebo (group 1)	GV1001 0.56 mg (group 2)	*P* value	GV1001 1.12 mg (group 3)	*P* value
		*n* = 26	*n* = 22		*n* = 25	
SIB	12	− 4.0 (1.4)	0.4 (1.5)	0.123	1.0 (1.4)*	0.016
	24	− 7.2 (1.9)	− 1.5 (2.0)	0.070	− 0.1 (1.9)*	0.018
K-MMSE	12	− 0.1 (0.5)	− 0.7 (0.6)	0.288	0.4 (0.6)	0.596
	24	− 0.7 (0.6)	− 1.1 (0.6)	0.446	− 0.2 (0.6)	0.575
CDR-SOB	12	0.5 (0.2)	0.4 (0.3)	0.829	0.4 (0.2)	0.739
	24	1.1 (0.3)	0.4 (0.4)	0.183	0.8 (0.3)	0.602
NPI	12	4.6 (2.6)	1.0 (2.7)	0.296	− 5.1 (2.6)*	0.017
	24	− 1.6 (4.3)	3.3 (4.4)	0.456	− 2.2 (4.4)	0.818
GDS	12	− 0.1 (0.1)	0.0 (0.1)	0.209	− 0.1 (0.1)	0.948
	24	− 0.1 (0.1)	0.0 (0.1)	0.073	− 0.1 (0.1)	0.733
ADCS-ADL	12	− 2.3 (0.8)	− 0.1 (0.9)	0.225	− 0.4 (0.8)	0.248
	24	− 4.0 (1.0)	− 2.4 (1.1)	0.516	− 2.7 (1.0)	0.470

*P* value for the differences between the treatment and placebo groups

The differences between the treatment group and placebo group were assessed using MMRM analysis

*Abbreviations*: *FAS* full analysis set, *PPS* per-protocol set, *LS* least square, *CFB* change from baseline, *SE* standard error, *SIB* Severe Impairment Battery, *K-MMSE* Korean Mini-Mental State Examination, *CDR-SOB* Clinical Dementia Rating Scale-Sum of Boxes, *NPI* Neuropsychiatric Inventory, *GDS* Global Deterioration Scale, *ADCS-ADL* Alzheimer’s Disease Cooperative Study-Activities of Daily Living, *MMRM* Mixed-effects Model Repeated Measures

*P < 0.05

GV1001 was confirmed to be as safe as normal saline (Table 3 and Table S7 in Additional file 2). There were no differences between the groups in terms of the most frequent treatment-emergent AEs (TEAEs) (Table S8 in Additional file 2). Two severe TEAEs occurred in the placebo group. One participant was hospitalized because of a fracture in one arm after sustaining a fall, and the other participant was incidentally diagnosed with stomach cancer (Table 3).

**Table 3:** Overall summary of treatment-emergent adverse events (TEAE) by severity: safety set population

All TEAEs	Placebo (group 1) (***n*** = 31)		GV1001 0.56 mg (group 2) (***n*** = 32)		GV1001 1.12 mg (group 3) (***n*** = 32)		Overall (***n*** = 95)		***P*** value
	***n*** (%)	Events	***n*** (%)	Events	***n*** (%)	Events	***n*** (%)	Events	
Mild	12 (38.7)	32	13 (40.6)	33	10 (31.3)	28	35 (36.8)	93	0.714^a^
Moderate	2 (6.5)	4	5 (15.6)	7	5 (15.6)	10	12 (12.6)	21	0.451^a^
Severe	2 (6.5)	2	0 (0.0)	0	0 (0.0)	0	2 (2.1)	2	0.104^b^

Note: study group 1 = placebo (control); study group 2 = GV1001 0.56 mg; study group 3 = GV1001 1.12 mg

*Abbreviations*: *TEAE* treatment-emergent adverse event, *n* number of patients

^a^Chi-square test; ^b^Fisher’s exact test

## Discussion

In this study, we demonstrated that GV1001 might have beneficial effects on the cognition and activities of daily living in patients with moderate-to-severe AD already receiving donepezil.

Since the approval of memantine, no drugs have been approved for AD. Although the FDA approval of aducanumab is awaited [7], certainly, there is a huge unmet medical need. Searching for additional pathomechanisms of AD and developing drugs with multifunctional effects are important. ROS resulting in oxidative stress, which is induced by aging, Aβ, and amyloid precursor protein, contributes to the pathogenesis of AD [26]. ROS may be involved in Aβ fibrillization in AD, which makes it a vicious cycle [26]. Neuroinflammation also plays a major role in the pathogenesis of AD. Although the inflammatory response may have beneficial effects by removing Aβ and tau, sustained inflammatory responses are detrimental [27]. At the early stage of Aβ pathology, Aβ is generally surrounded by the neuroprotective phenotype of microglia [28]. As the disease progresses, the microglia switch to a more neurotoxic phenotype [27, 28]. In our previous study, GV1001 restricted the production of ROS, which was increased by Aβ oligomer [14]. Moreover, GV1001 decreased death and inflammation-related proteins, which were increased by Aβ or oxidative stress [14, 15]. In addition to its anti-oxidant and anti-inflammatory effects, GV1001 showed some other beneficial effects, such as anti-aging and mitochondria-stabilizing effects against Aβ toxicity and other stressors [14–16]. GV1001 effectively entered the brain through the brain-blood barrier, which was confirmed in the MRI and Prussian blue staining studies detecting subcutaneously injected GV1001 labeled with ferrocenecarboxylic acid (Fe) (Additional file 2, Figure S1). Hence, we hypothesized that GV1001 might be helpful in the treatment of patients with AD.

This clinical trial showed that GV1001, especially at a dose of 1.12 mg, effectively reduced the change in SIB scores compared with the placebo treatment, suggesting that GV1001 might have beneficial effects in patients with moderate-to-severe AD. We assessed the change in the SIB score from baseline to week 24 as a primary endpoint because SIB is suitable for evaluating patients with moderate-to-severe AD as it overcomes the floor effects of patients with more advanced disease [29]. In our control group, the SIB score significantly decreased from baseline to week 24; however, this decrease was consistent with that reported in previously published clinical trials [30, 31]. In a previous study including patients with moderate-to-severe AD, the decrease in the SIB score was > 10 in the control group 28 weeks after initiation of the trial [30]. In another study, the SIB score was 6.6 in the control group after 24 weeks of applying a rivastigmine patch (4.6 mg/24 h) [31], although this decrease was significantly higher than the approximate SIB score of 2 at 24 weeks reported in several other studies [32, 33].

As the other primary endpoint, we assessed the safety of GV1001. There was no difference in the TEAEs between the three groups; additionally, no severe TEAEs were noted in the GV1001 groups, regardless of the dose (Table 3 and Tables S7 and S8 in Additional file 2), suggesting that the administration of GV1001 0.56 and 1.12 mg is as safe as the administration of normal saline. This finding will make it easier for us to perform subsequent clinical trials. The number of dropouts was not significantly different among the three groups (5 in group 1, 10 in group 2, and 7 in group 3, *p* = 0.356). Participants from a single center were excluded after the administration of the first study treatment, due to mandatory clinical data, including baseline clinical data and the results of neurological examination performed at the corresponding time points, were not uploaded to the central web-based system and the investigator of this center could not verify the source of these data (Fig. 2). The exclusion resulted in a slightly higher number of dropouts in group 2. Nevertheless, this exclusion occurred randomly as the allocation of participants from this center occurred randomly by the Interactive Web Response System and there was no significant difference in the baseline clinical characteristics among patients who dropped out.

Regarding the secondary endpoints, the pattern of the changes from the baseline in ADCS-ADL and CDR-SOB were similar to that of the primary endpoint; however, the change was not statistically significant (Table 2 and Fig. 3). The relatively small number of subjects might have restricted us from showing statistical significance. The NPI scores showed a statistically significant improvement at week 12 in the GV1001 1.12 mg group, even though the significance was not maintained at week 24 (Table 2). There were no significant differences in other secondary endpoints between the GV1001 groups and control group. The effect of GV1001 on the secondary endpoints needs to be confirmed in a larger clinical trial because our study was performed to investigate the feasibility of GV1001 for the treatment of moderate-to-severe AD, and the size of each group was small.

This study has merit in that significant benefit was observed for GV1001 at both weeks 12 and 24 with a relatively small number of patients with moderate-to-severe AD. The diverse neuroprotective effects of GV1001, such as its anti-inflammatory, anti-aging, antioxidant, and mitochondria-stabilizing effects, might reveal these results. However, further research is needed to show the association between these neuroprotective effects and biomarkers of AD. The incidence of dropout and AEs were comparable among the three groups.

The limitations of this study include the enrollment of a single ethnic population and short duration of follow-up. However, as we enrolled elderly and vulnerable (moderate-to-severe AD) patients, the concern about high attrition rates restrained us from planning a longer study period (≥ 12 months). For this reason, several previous randomized controlled trials involving moderate-to-severe AD observed participants for approximately 6 months [30, 34]. When we decided to enroll patients with moderate-to-severe AD, we considered our in vivo study results (unpublished data) showing that GV1001 significantly improved the neurobehavioral functions of old (from the age of 21 months until the endpoint) 3xTg-AD mice (B6:129-Psen1tm1Mpm Tg[APPSwe, tauP301L]1Lfa/Mmjax), which are comparable to patients with moderate-to-severe AD. As we enrolled patients with moderate-to-severe AD, a group receiving only GV1001 without donepezil was not evaluated in terms of ethical problems. Moreover, drugs other than donepezil (i.e., memantine) for the treatment of AD were prohibited because it would be difficult to show the effectiveness of the drug in the first human clinical with a small number. Additionally, we could not include pharmacokinetic (PK) data, as the detection level of GV1001 in the blood was extremely low to show a sufficient PK profile in humans. The peptides may rapidly degrade before their uptake by antigen-presenting cells [35]. The doses of the investigational drug to be used in this study were determined by the process for deriving the maximum recommended starting dose for the first-in-human clinical trials of new molecular entities and preclinical experiments in an AD mouse model. Finally, biomarkers associated with AD were not investigated as a part of AD diagnosis because the diagnosis was based on DSM-IV and the 2011 recommendations from the National Institute on Aging-Alzheimer’s Association workgroups on diagnostic guidelines for Alzheimer’s disease, which did not recommend any biomarker studies for the diagnosis of moderate to severe AD [17]. Moreover, this clinical trial had been planned before the 2018 National Institute on Aging-Alzheimer’s Association Research Framework was published [36], so we did not consider any biomarker studies in this trial. Nevertheless, this might have biased the diagnosis of AD in a few patients. Therefore, to overcome this bias, additional biomarkers, including neurodegeneration biomarker (hippocampal volume or cortical thickness), Aβ positivity, and apolipoprotein E genotyping, should be considered in future larger clinical trials. Evaluating the level of neurofilament light or tau in blood or cerebrospinal fluid can be also helpful in proving the biological effect of GV1001.

## Conclusions

This phase 2 trial showed that GV1001 may provide beneficial effects without safety concerns in patients with moderate-to-severe AD. GV1001 1.12 mg met the primary endpoint, and this study warrants a larger clinical trial. The following clinical trials should consider evaluating additional biomarkers, considering other drugs for the treatment of AD, analyzing sample size based on this study, and including diverse ethnic populations. Based on these results, another phase 2a clinical trial for moderate AD has already been approved in the USA after a thorough review by the FDA (NCT03959553 for ClinicalTrials.gov and IND 137519 for FDA), and a phase 3 clinical trial in South Korea will be performed soon.

## Supplementary Information

**Additional file 1.** Clinical trial protocol.

**Additional file 2: Table S1.** Inclusion and exclusion criteria of this clinical trial. **Table S2.** Participant number of each group. **Table S3.** Observed mean total score of each outcome. **Table S4.** Summary of CIBIS and CIBIC-plus distributions in full analysis set. **Table S5.** Least mean difference from placebo in full analysis set. **Table S6.** Least mean difference from placebo in per-protocol population. **Table S7.** Overall summary of treatment-emergent adverse events: safety set population. **Table S8.** Most frequent treatment-emergent adverse events that occurred in > 2 patient overall: safety set population. **Figure S1.** GV1001 (1 mg/kg) or an equivalent volume of 0.9% saline was subcutaneously injected into old 3xTg-AD mice (B6:129-Psen1tm1Mpm Tg[APPSwe, tauP301L]1Lfa/Mmjax) from the age of 21 months until the mice were deemed ready for sacrifice according to the CCAC guidelines on selecting an appropriate endpoint in experiments using animals for research, teaching, and testing. The injections were administered three times a week until the endpoint. The neurobehavioral functions were evaluated every 3 days until the endpoint using the Y-maze test and passive avoidance task. A. In the Y-maze test, which was used to measure the willingness of rodents to explore new environments and therefore quantify the cognitive deficits,^1,2^ compared with 0.9% saline, 1 mg/kg GV1001 significantly improved the percentage of spontaneous alternations among the Y-maze arms. B and C. In the passive avoidance tasks, which is a fear-aggravated test used to evaluate learning and memory, the mice received an electric shock when they entered the dark compartment^3,4^; therefore, the latency to enter the dark compartment and number of errors reflected the learning and memory of the mice. The latency to enter the dark compartment and number of errors were significantly improved following treatment with 1 mg/kg GV1001 (B and C, respectively). D. To confirm that GV1001 can enter the brain, 1 mg/kg of GV1001 conjugated with ferrocenecarboxylic acid, which we used in our previous study,^5^ was subcutaneously injected into 3xTg-AD (12-month-old) mice. It was detected as dark signals in the brain using 3 T magnetic resonance imaging (white arrows) and Prussian blue staining (black boxes).

## Data Availability

Access to participant-level data from this study will not be made available, while GV1001 is in clinical trials for Alzheimer’s disease and waiting for approval from the US Food and Drug Administration. Thereafter, all data supporting this study will be shared by qualified academic researchers after obtaining the consent of researchers.

## Abbreviations

ADCS-ADL: Alzheimer’s Disease Cooperative Study-Activities of Daily Living
AEs: Adverse events
CDR-SB: Clinical Dementia Rating Sum of Box
CIBIC-plus: Clinician Interview-Based Impression of Change plus caregiver input
FAS: Full analysis set
GDS: Global Deterioration Scale
MMSE: Mini-Mental State Examination
NPI: Neuropsychiatric Inventory
PPS: Per-protocol set
SC: Subcutaneous
SIB: Severe Impairment Battery
TEAEs: Treatment-emergent AEs
