# Supplementary material for: Efficacy and safety of GV1001 in patients with moderate-to-severe Alzheimer’s disease already receiving donepezil: a phase 2 randomized, double-blind, placebo-controlled, multicenter clinical trial
Source: Alzheimers Res Ther. 2021 Mar 26;13:66. doi: 10.1186/s13195-021-00803-w (PMC7995588; doi:10.1186/s13195-021-00803-w)
Supplement: Supplementary file 1 — Additional file 1. Clinical trial protocol. [file 13195_2021_803_MOESM1_ESM.pdf]

CLINICAL TRIAL PROTOCOL

A Multi-center, Randomized, Double-blind, Placebo-controlled, Parallel Design, Prospective, Phase II Clinical Trial to Evaluate the Efficacy and Safety of Subcutaneous Administration of GV1001 0.56 mg/day and 1.12 mg/day in Combination with Donepezil in Alzheimer Patients

**Clinical Trial Protocol No.:**	**KG6/2016**
**Clinical Trial Protocol Version:**	**Version 3.0**
**Date of Issue:**	**2017.05.02**

CONFIDENTIAL

Any and all information included in this clinical trial protocol should be provided only for those involved in this clinical trial including the principal investigator, the subinvestigator, the Institutional Review Board (IRB), and the Ministry of Food and Drug Safety. It should not be open to a third party without prior written consent of GemVax & KAEL Co., Ltd., except for when the investigator has to obtain informed consent to participate in this clinical trial from patients who will be treated with the investigational product used for this trial.

TABLE OF CONTENTS

▣ DEFINITION OF ABBREVIATIONS AND TERMS 10

▣ SYNOPSIS 12

▣ TRIAL FLOW CHART... 20

1. TITLE AND PHASE OF CLINICAL TRIAL.. 22

1.1. Title.. 22

1.2. Phase 22

2. NAME AND ADDRESS OF STUDY CENTERS 22

3. NAME AND POSITION OF PRINCIPAL INVESTIGATOR, SUBINVESTIGATOR AND CO-INVESTIGATOR.. 22

3.1. Subinvestigator and CRP 22

4. NAME AND ADDRESS OF SPONSOR 22

4.1. Name and Address of Sponsor 22

5. INTRODUCTION 22

5.1. Background of Clinical Trial 22

5.1.1. Alzheimer's disease 22

5.1.2. GV1001 and AD 24

5.1.3. GV1001's mechanism of action 30

5.2. Necessity of Clinical Trial 31

6. OBJECTIVE OF CLINICAL TRIAL 32

6.1. Primary Efficacy Endpoint 32

6.2. Secondary Efficacy Endpoint 32

6.3. Safety Endpoint 33

7. INVESTIGATIONAL PRODUCTS USED IN CLINICAL TRIAL 33

7.1. Outline of Investigational Products 33

7.1.1. Investigational products (group1) 33

7.1.2. Investigational products (group2) 33

7.1.3. Investigational Reference Product(s) 34

7.2. Amount, Route and Duration of Administration 34
7.2.1. Amount and Route of Administration 34
7.2.2. Duration of Administration 34
7.3. Production, Packaging and Labeling of the Investigational Product . 35
7.4. Management of Investigational Product 36
7.5. Maintenance of Blinding and Unblinding 36
7.6. Concomitant Medications and Therapy/Prohibited Concomitant Medications and Therapy 37
7.6.1. Allowed concomitant medications 37
7.6.2. Prohibited concomitant medications and therapy 37
7.6.3. Drugs requiring caution 38
8. TARGET DISEASE 39
9. INCLUSION CRITERIA, EXCLUSION CRITERIA, TARGET SAMPLE SIZE AND JUSTIFICATION 39
9.1. Inclusion Criteria 39

9.2. Exclusion Criteria 40

9.3. Justification for Sample Size Calculation 41

10. STUDY PERIOD 42

11. STUDY METHOD 42

11.1. Design 42

11.2. Assignment to Administration Groups 43

12. OBSERVATION ITEMS, CLINICAL EXAMINATION ITEMS AND OBSERVATIONAL EXAMINATION METHODS 43

12.1. Evaluation Method of Clinical Trial 43

12.1.1. Efficacy evaluation 43

12.1.2. Safety evaluation 46

12.1.3. Others 49

12.2. Per-visit Procedures 49

12.2.1. Visit 1 (within -2 weeks; screening) 49

12.2.2. Visit 2 (1 week: Randomization and Administration of the Investigational Product) ... 50

12.2.3. Visits 3~5 (2, 3, and 4 weeks ± 2 days ; Administration of the Investigational Product) ... 51

12.2.4. Visits 6~8 (6, 8, and 10 weeks ± 3 days ; Administration of the Investigational Product) ... 51

12.2.5. Visit 9 (12 weeks ± 3 days ; Administration of the Investigational Product) ... 52

12.2.6. Visits 10~14 (14, 16, 18, 20, and 22 weeks ± 3 days ; Administration of the Investigational Product) ... 52

12.2.7. Visit 15 (24 weeks ± 3 days ; Administration of the Investigational Product) ... 53

12.2.8. Visit 16 (28 weeks ± 3 days; End of Trial) ... 53

12.2.9. Additional visits ... 54

13. PREDICTED ADVERSE EVENTS AND PRECAUTIONS ... 54

14. CRITERIA OF TRIAL DISCONTINUATION AND DROPOUTS ... 55

14.1. Criteria of Trial Discontinuation and Dropouts 55

14.2. Treatment After the Termination of Clinical Trial.............................. 56

15. STATISTICAL ANALYSIS METHOD 57

15.1. Statistical Analysis Plan.. 57

15.1.1. Definition of Analysis Set 57

15.1.2. General principle of statistics 57

15.1.3. Analysis of Demographic Data and Baseline Features Data...... 57

15.1.4. Efficacy analysis 58

15.1.5. Safety analysis 59

15.1.6. Handling of dropouts or missing values....................................... 60

15.1.7. Handling of protocol violations 61

15.1.8. Scheduled interim analysis and data monitoring 61

16. SAFETY EVALUATION CRITERIA, EVALUATION METHODS AND REPORT METHODS INCLUDING ADVERSE EVENTS...................................... 62

16.1. Definition of Adverse Events 62

16.2. Collection and Recording of Adverse Events 63

16.3. Evaluation of Adverse Events 63

16.3.1. Severity evaluation 63

16.3.2. Evaluation of causal relationship 64

16.3.3. Determination of whether an AE is unexpected 65

16.4. Reporting of Serious Adverse Events 66

16.5. Pregnancy 68

17. OTHER MATTERS TO CONDUCT THIS TRIAL IN A SAFE AND SCIENTIFIC MANNER 68

17.1. Agreement and Compliance with the Clinical Trial Protocol 68

17.2. Approval and Amendment of Clinical Trial Protocol 68

17.3. Consent Procedure of Patients 68

17.4. Measures for Protection of Patient's Safety 69

17.5. Medical Treatment and Medical Treatment Standards for Patients After Clinical Trial 69

17.6. Patient Compensation Regulation 70

17.7. Clinical Trial Documents and Preservation of Documents 70

17.7.1. CRF and source documents 70

17.7.2. Accessibility to source documents 70

17.7.3. Preservation of clinical trial documents 70

17.7.4. Audit & Inspection 71

17.8. Confidentiality of Clinical Trial Documents and Patient Records 71

17.9. Monitoring of Study Center 71

17.10. Discontinuation of Clinical Trial 72

17.11. Reporting and Publishing of Clinical Trial Results 72

18. REFERENCES 72

19. ANNEX LIST 74

▣ DEFINITION OF ABBREVIATIONS AND TERMS

AD	: Alzheimer's Disease
ADCS-ADL-severe	: Alzheimer's Disease Cooperative Study-Activities of Daily Living scale-severe
ADL	: Activities of Daily Living
ADR	: Adverse Drug Reaction
AE	: Adverse Event
ALT	: Alanine Aminotransferase
ALP	: Alkaline Phosphatase
AST	: Aspartate Aminotransferase
BUN	: Blood Urea Nitrogen
CIBIC-Plus	: Clinician Interview-Based Impression of Change-Plus
CDR-SOB	: Clinical Dementia Rating-Sum of Box
CES-D	: Centre for Epidemiological Studies - Depression Scale
CGI	: Clinical Global Impression
CRPS	: Complex Regional Pain Syndrome
DPRS	: Daily Pain Rating Scale
DSIS	: Daily Sleep Interference Scale
DSM-IV	: Diagnostic and Statistical Manual of Mental Disorders, Fourth Edition
ECG	: Electrocardiography
FAS	: Full Analysis Set
FDA	: Food and Drug Administration
F/U	: Follow Up
GDS	: Global Deterioration Scale
HDL	: High-Density lipoprotein
HIV	: Human Immunodeficiency Virus
K-MMSE	: Korean-Mini-Mental State Examination
LDH	: Lactate Dehydrogenase
LDL	: Low-Density Lipoprotein
NINDS-ADRDA	: National Institute of Neurological Disorders and Stroke and the Alzheimer's Disease and Related Disorders Association
NPI	: Neuropsychiatric Inventory
PGI	: Patient Global Impression
pH	: Negative Logarithm of the Hydrogen Ion Concentration
PPS	: Per Protocol Set
RBC	: Red Blood Cell

SAS	: Statistical Analysis Software
SGOT	: Serum Glutamic Oxaloacetic Transaminase
SGPT	: Serum Glutamic Pyruvic Transaminase
SIB	: Severe Impairment Battery
SUSAR	: Suspected Unexpected Serious Adverse Reaction
WBC	: White Blood Cell
γ-GTP	: Gamma-Glutamyl Transpeptidase

▣ Synopsis

**TITLE OF CLINICAL TRIAL**	A multi-center, randomized, double-blind, placebo-controlled, parallel design, prospective, Phase II clinical trial to evaluate the efficacy and safety of subcutaneous administration of GV1001 0.56 mg/day and 1.12 mg/day in combination with donepezil in Alzheimer patients.
**Sponsor**	GemVax & KAEL Co., Ltd. CEO: Sang Jae Kim
**Contract Research Organization (CRO)**	CliPS Co., Ltd. CEO Jun Hwan Jee<br>13F, Police Mutual Aid Association Jaram Building, 78, Mapo-daero, Mapo-gu, Seoul, Korea
**Study Center and Principal Investigator**	Professor Seong Ho Koh<br>Department of Neurology, Hanyang University Guri Hospital
**Study Period**	24 months from the IRB approval date
**Target Disease**	Moderate to severe Alzheimer's disease
**Objective of Clinical Trial**	This clinical trial, as a therapeutic exploratory clinical trial for regulating severity of disease, controlling progress of disease, alleviating symptoms, and setting mono-therapy or additive therapy of donepezil when GV1001 manufactured by GemVax & KAEL Co., Ltd. is subcutaneously administered to Alzheimer patients at a dose of 0.56 or 1.12 mg/day, evaluates efficacy and safety in GV1001 0.56 mg group and GV1001 1.12 mg group for moderate to severe Alzheimer patients.
**Phase and Design of Clinical Trial**	A multi-center, randomized, double-blind, placebo-controlled, parallel design, prospective phase II clinical trial
**Trial Method**	This clinical trial was designed as a multi-center, randomized, double-blind, placebo-controlled, parallel design, prospective phase II clinical trial.<br><br>If a patient or a legally acceptable representative agrees in writing to participate in this clinical trial at the screening visit, the necessary checkup and examination are carried out during the screening visit in accordance with the clinical trial protocol and the screening lasts within 2 weeks.

▣ Synopsis

	For patients considered fit for the inclusion criteria as a result of evaluating the suitability as patients of this trial after the completion of screening, they are assigned to treatment group 1(GV1001 0.56 mg), treatment group 2 (GV1001 1.12 mg), or control group (placebo) in the ratio of 1 to 1 to 1; and are treated with the investigational product or placebo 4 times at intervals of 1 week and then 10 times at intervals of 2 weeks (a total of 14 times) to evaluate their safety and efficacy.<br><br>In order to ensure the objectivity and accuracy of trial results, efficacy evaluators should be limited to those who have sufficiently received education and training, and efficacy evaluation per visit should be conducted in a fixed order.<br><br>Donepezil, which has been stably administered for 3 months or more before screening, can be administered without changes in dosage over the duration of clinical trial.<br>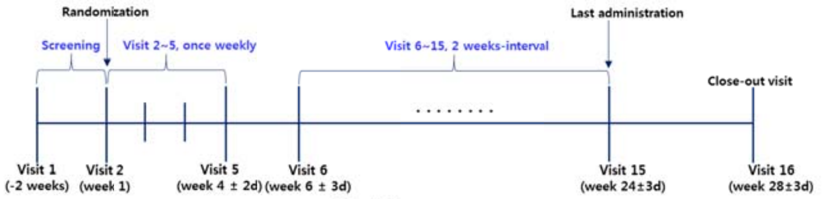<br>[Study Flow]
**Investigational Product**	• Investigational product: GV1001 0.56mg, 1.12mg<br>• Investigational reference product: Placebo of GV1001
**Administration and Dosage**	The investigational product and placebo are subcutaneously administered once weekly for 4 weeks and then every 2 weeks through Week 24 (a total of 14 times) in accordance with the schedule specified in the clinical trial protocol.
**Study Period**	About 24 months after the IRB approval

▣ Synopsis

<table>
<tr><td>Sample Size</td><td>This clinical trial is designed to evaluate the ability of GV1001 to reduce disease progression and to assess itys safety profile in moderate to severe Alzheimer patients; and a total of 90 patients (sample size eligible for efficacy evaluation: 72) will be collected from 12 institutions.
<table>
<tr><th></th><th>Treatment Group 1</th><th>Treatment Group 2</th><th>Control group</th><th>Total sample size</th></tr>
<tr><td>Sample size for efficacy evaluation</td><td>24</td><td>24</td><td>24</td><td>72</td></tr>
<tr><td>Sample size including dropout rate (20%)</td><td>30</td><td>30</td><td>30</td><td>90</td></tr>
</table>
</td></tr>
<tr><td>Inclusion Criteria</td><td>1) Aged ≥ 55 to ≤ 85 years, inclusive, at the time of signing the informed consent.
2) Meet the Diagnostic and Statistical Manual of Mental Disorders, fourth edition (DSM-IV) criteria for diagnosing dementia.
3) Clinically diagnosed with probable AD as defined in the National Institute of Neurological and Communicative Disorders and Stroke (NINCDS) and the Alzheimer’s Disease and Related Disorders Association (ADRDA) criteria.
4) Korean Mini-Mental Status Examination score ≤ 19 at the screening visit.
5) Rated as Grade 5 to 6 on the GDS.
6) Have no other diseases to cause dementias other than AD as a result of an MRI or CT scan within 12 months prior to the screening visit.
7) Received donepezil at stable doses for 3 months or more prior to the screening visit.
8) Able to undergo cognitive and other tests by walking on their own or visiting hospitals using an assist device on an outpatient basis or for hospitalization.
9) Have a guardian who is able to accompany the patient for all visits, supervise the patient's compliance with the procedures specified in the clinical trial protocol and the investigational product, and provide detailed patient information.
10) Voluntarily agreed to participate in this clinical trial and signed the</td></tr>
</table>

▣ Synopsis

	patient consent form (if there is no legally authorized representative, the role should be assumed in the order of a spouse, a lineal ascendant, and a direct descendant. If there are several lineal ascendants or direct descendants, consultation is needed, and if no agreement is reached, the oldest should become a legally authorized representative*.)<br>* Paragraph 2, Article 16 of the Act on Bioethics and Safety.
**Exclusion Criteria**	1) Diagnosis of other causes of dementia as listed below as a result of a CT/MRI test and neurologic examination within 12 months prior to screening or at the screening visit:<br>- Possible, probable or definite vascular dementia according to the National Institute of Neurological Disorders and Stroke and the Association Internationale pour la Recherche et l'Enseignement en Neurosciences (NINDS AIREN) criteria.<br>- Other central nervous system diseases that may cause the impairment of cognitive function (cerebrovascular disease including cerebrovascular dementia, Parkinson's disease, Huntington's disease, subdural hematoma, normal pressure hydrocephalus, brain tumor, Creutzfeldt-Jakob disease).<br>- Neuropathy such as delusion, delirium, epilepsy.<br>2) Abnormal laboratory test results which are considered to contribute to the severity of their dementia or are a cause of dementia e.g. vitamin B12/folic acid levels, abnormal syphilis serology, and thyroid stimulating hormone (TSH) levels.<br>3) History of depression or a history of significant psychiatric illness which according to the investigator's judgment may interfere with the participation of this clinical trial, such as schizophrenia or bipolar affective disorders.<br>4) History of known or suspected seizures, including febrile seizure, or recent unexplained loss of consciousness, or a history of significant head trauma accompanied by loss of consciousness.<br>5) Acute or unstable cardiovascular disease, active peptic ulcer, or uncontrolled hypertension, uncontrolled diabetes or insulin dependent patients, or any medical condition that may interfere with the completion of the clinical study.

▣ Synopsis

	6) History of hypersensitivity to GV1001 or to medicinal products with similar chemical structures.<br>7) History of alcohol and drug abuse or dependence (except nicotine dependence) within the last 2 years.<br>8) History of cancer within the past 5 years, except non-metastatic skin basal cell carcinoma and/or skin squamous cell carcinoma, carcinoma in situ of uterine cervix, or non-progressive prostate cancer.<br>9) Renal dysfunction (define as creatinine clearance [CLcr] < 30 mL/min).<br>10) Serious hepatic dysfunction (define as ALT or AST≥ 2.0 times the upper limit of normal).<br>11) Administered drugs other than donepezil to treat AD or other cognitive function impairments.<br>12) Prohibited medications and therapies specified in the Clinical Protocol, Section 7.6.2, such as anticholinergic drugs, choline excitatory drugs antidepressant drugs (tricyclic antidepressants, monoamine oxidase inhibitors), typical antipsychotic drugs, central stimulant, and other drugs for AD other than donepezil or who are scheduled to receive them over the duration of this clinical trial.<br>*Note: Drugs for local administration such as pilocarpine collyrium are permitted.*<br>13) Women of reproductive age without a negative pregnancy test and without a commitment to using an acceptable method of contraception (e.g., condoms, diaphragms, oral contraceptives and long acting progestin agents), if sexually active, until the end of the trial. Women who are postmenopausal (1 year since last their menstrual cycle), surgically sterilised or who have undergone a hysterectomy are considered not to be reproductive and can be included.<br>14) Pregnant or lactating women.<br>15) Participated in a clinical study with any experimental treatment within 4 weeks prior to the screening visit or previous participation in the present study.<br>16) Weight ≤ 35 kg at screening and admission visits.

▣ Synopsis

	17) Previous treatment with the investigational product.<br>18) Participated in Alzheimer's-type dementia vaccine clinical trial (e.g.: amyloid vaccine) within 6 months prior to the clinical trial.<br>19) Any other condition that in the opinion of the investigator can interfered with the interpretation of the study results or constituted a health risk for the patient if he/she take part in the study.
**Efficacy Evaluation**	**Primary Efficacy Variable:**<br>Change from baseline in SIB score at Visit 15 (2Week 24)<br>**Secondary Efficacy Variables:**<br>1) Change from baseline in Korean Mini-Mental State Examination (K-MMSE) score at Visit 15 (Week 24).<br>2) Change from baseline in Clinical Dementia Rating-Sum of Box (CDR-SOB) score at Visit 15 (Week 24).<br>3) Change from baseline in Neuropsychiatric Inventory (NPI) score at Visit 15 (Week 24).<br>4) Change from baseline in Global Deterioration Scale (GDS) score at Visit 15 (Week 24).<br>5) Change from baseline in Alzheimer's Disease Cooperative Study-Activities of Daily Living-severe (ADCS-ADL-severe) score at Visit 15 (Week 24).<br>6) Change from baseline in Clinician Interview-Based Impression of Change-Plus (CIBIC-plus) score at Visit 15 (Week 24)
**Safety Evaluation**	• Adverse events<br>• Laboratory tests (Hematological tests, Blood Chemistry, Urinalysis)<br>• Vital signs
**Statistical Methods**	**1. Evaluation of efficacy**<br>**1) Primary efficacy variable**<br>For SIB variations at end point (24 weeks) compared to the baseline between treatment group and control group at each dose, the mean, standard deviation, minimum value, and maximum value will be presented; and whether there are differences in variation at the baseline and at the end point between treatment group and control group at each dose will be examined through the t-test or the Wilcoxon's rank sum test according to the layered testing strategy.

▣ Synopsis

	**2) Secondary efficacy variables**<br>For K-MMSE, CDR-SOB, NPI, GDS, ADCS-ADL-severe variations at the time point of 24 weeks compared to the baseline between treatment group and control group at each dose, the mean, standard deviation, minimum value, and maximum value will be presented; and whether there are differences in variation at the baseline and at the time point of 24 weeks between treatment group and control group at each dose will be examined through the t-test or the Wilcoxon's rank sum test according to the layered testing strategy. CIBIC-plus will be examined though the chi-square test.<br><br>**2. Evaluation of Safety**<br>After all AEs reported over the duration of clinical trial are charted, the ratio of the patients, who experienced AEs and SAEs once or more among those who have been administered the investigational product at least once between groups, will be presented. Whether there is any significant difference between groups will be compared and analyzed using the Pearson's chi-square test or the Fisher's exact test.<br><br>For the clinical laboratory tests, vital signs and physical examinations, intragroup and intergroup comparisons will be carried out.<br><br>- Intragroup comparisons: For continuous variables, the descriptive statistics describing baseline, value at end time, and difference between baseline and end time will be presented; and the mean variation is analyzed using the paired t-test or the Wilcoxon's signed rank test. For categorical variables, a contingency table will be completed, and it will be analyzed using the McNemar's test.<br><br>- Intergroup comparisons: For continuous variables, difference between baseline and end time will be compared and analyzed using the ANOVA or the Kruskal-Wallis test. For categorical variables,

▣ Synopsis

	homogeneity between treatment groups will be analyzed using the Pearson's chi-square test, the Fisher's exact test, etc.

▣ TRIAL FLOW CHART

	Screening	Duration of Treatment														End of Trial
Visit	V1	V2	V3	V4	V5	V6	V7	V8	V9	V10	V11	V12	V13	V14	V15	V16
Week	≤ -2 weeks	W1	W2	W3	W4	W6	W8	W10	W12	W14	W16	W18	W20	W22	W24	W28
Visit window			±2	±2	±2	±3	±3	±3	±3	±3	±3	±3	±3	±3	±3	±3
Patient consent consent and provision of screening number	O															
Checking the inclusion/exclusion criteria	O	O														
Randomization and provision of registration number		O														
Demographic information	O															
Checking medical history	O	O														
Height	O															
Body weight	O	O	O	O	O	O	O	O	O	O	O	O	O	O	O	O
Vital signs	O	O	O	O	O	O	O	O	O	O	O	O	O	O	O	O
Physical examination	O	O	O	O	O	O	O	O	O	O	O	O	O	O	O	O
ECG	O															
Checking previous /concomitant medications	O	O	O	O	O	O	O	O	O	O	O	O	O	O	O	O
SIB		O							O						O	
K-MMSE[1]	O								O						O	
GDS[1]	O								O						O	
CIBIC Plus		O							O						O	
NPI		O							O						O	
CDR-SOB		O							O						O	
ADCS-ADL-severe		O							O						O	
Laboratory tests[2]	O								O						O	
Administration of investigational product or placebo[3]		O	O	O	O	O	O	O	O	O	O	O	O	O	O	

Evaluation of AEs		O[7]	O	O	O	O	O	O	O	O	O	O	O	O	O	O
Urine hCG test[4]	O														O	
CLcr test[5]	O															
CT/MRI[6]	O															

[1] The result of V1 is used as baseline. However, if there is a result of the test conducted at the relevant institution within 2 weeks, it can be replaced with this result.

[2] If there is a result of the test conducted at the relevant institution within 4 weeks based on the date of screening, it can be replaced with this result.

Hematological tests: WBC, RBC, Hemoglobin, Hematocrit, Platelets count, WBC Diffcount (Neutrophils, Lymphocytes, Monocytes, Eosinophils, Basophils)

Blood Chemistry: BUN, Creatinine, Uric acid, Total bilirubin, Albumin, Total Protein, ALT, AST, γ-GTP, Alkaline phosphatase, Glucose, Total Cholesterol

* Only during screening visits: Vitamin B12, Folic acid, HbA1c

Urinalysis: Protein (Albumin), Glucose, Ketones, WBC, Blood (RBC)

Blood coagulation test: INR (Only during screening visits)

Thyroid function tests: TSH, free T4 (Only during screening visits)

Syphilis, AIDS tests: VDRL, anti-HIV (Only during screening visits)

[3] Administration of investigational product or placebo: Observation is made for 30 minutes after the administration of the investigational product at V2~V15.

[4] Urine hCG test is performed only for women of childbearing age except for women who have undergone a sterilization operation or who have reached menopause.

[5] Creatinine clearance test: CLcr (Only during screening visits)

[6] CT/MRI test: If there are results of the tests (CT, MRI) conducted within 12 months based on the date of screening, it can be replaced with these results. (Only during screening visits)

[7] At Visit 2, the assessment of AEs refers to AEs observed for 30 minutes after the administration of the investigational product.

1. TITLE AND PHASE OF CLINICAL TRIAL

1.1. Title

A Multi-center, Randomized, Double-blind, Placebo-controlled, Parallel Design, Prospective, Phase II Clinical Trial to Evaluate the Efficacy and Safety of Subcutaneous Administration of GV1001 0.56 mg/day and 1.12 mg/day in Combination with Donepezil in Alzheimer Patients

1.2. Phase

Phase II Clinical Trial

2. NAME AND ADDRESS OF STUDY CENTERS

The list of study centers is managed separately as the list of study centers and investigators. (Refer to Annex 1.)

3. NAME AND POSITION OF PRINCIPAL INVESTIGATOR, SUBINVESTIGATOR AND CO-INVESTIGATOR

3.1. Subinvestigator and CRP

Subinvestigator and Clinical Research Pharmacist (CRP) are separately managed. (Refer to Annex 1.)

4. NAME AND ADDRESS OF SPONSOR

4.1. Name and Address of Sponsor

GemVax & KAEL Co., Ltd. C.E.O: Sang Jae Kim

Address: 117, Unjung-ro, Bundang-gu, Gyeonggi-do, Korea

5. INTRODUCTION

5.1. Background of Clinical Trial

5.1.1. Alzheimer's disease

Alzheimer's disease (AD) is a disease which causes dementia by the impairment of neuron functions and reduction of neurons resulting from damaged communications, inflammatory response, brain cell death, etc. between neurons and glial cells due to neurotoxicity driven by the condensation and deposition of amyloid-β 40-42 protein and tau protein which are specific proteins. It is a syndrome that considerably worsens cognitive and memory functions including speech, visual perception, space organization and management abilities, etc. as well as chronic memory loss which is a serious disturbance to daily life. Thus, AD is characterized by gradual decreases in interpersonal relations, occupational function, and daily living function. AD is estimated to account for 60 to 80% of dementias.

According to a report by Alzheimer's Disease International, the worldwide federation of Alzheimer associations consisting of six countries, the number of dementia patients around the world is estimated at 44 million as of 2013; and it is expected the number will reach 76 million and 135 million by 2030 and 2050, respectively. The biggest reason for this is cited as aging population. In Korea, the statistics of the Korea Health Industry Development Institute show that the prevalence rate of dementias (number of domestic elderly people with dementia aged ≥65) is 8.4% as of the first half of 2013, and the prevalence rate is estimated to be 13.2% by 2050. This increase in prevalence due to the rapid increase in the elderly population has become a social problem because it brings great financial and psychological burdens to the patient as well as the family and society.

Although there have been many studies to develop drugs for inhibiting the progress of AD as well as to clearly define the etiological causes around the world, the causes have not been yet definitely revealed.

However, according to the result of researches accumulated so far, the major cause of AD is judged pathologically to be the infiltration into the outside of neurons by β amyloid plaque composed of abnormal amyloid proteins, killed neurons and inflammatory cells; and the condensation of neurofibrillary tangle (NFT) composed of Hyperphosphorylated tau proteins, which was detected inside neurons.

Neurodegeneration in AD is characterized at the early stage by abnormal transmission and metabolism of neurotransmitters, extracellular β-amyloid (Aβ) accumulation (amyloid/senile plaques) and intracellular neurofibril knots (NFTs), gliosis and inflammatory response, followed by brain cell toxicity due to accumulated β-amyloid and NFT, which leads to brain cell death. Latterly, considerable neuron loss and brain atrophy associated with this occur. In early days, the effects are limited to entorhinal cortex and hippocampus, and cognitive and memory functions are impaired. As AD progresses, it affects the generation and death of the brain's synapses which form new memories, resulting in the death of nerves up to 80% of hippocampus. As symptoms of AD progress, cognitive disorders and disturbance of daily living occur and the overall clinical global impression score worsens.

AD's molecular biological pathogenesis is that because of protein misfolding, amyloid-β and tau protein form and accumulate plaques and tangles respectively, leading to brain cell death. Amyloid plaques, which are made up of the amyloid-β fragments cut by β- secretase and γ-secretase in APP, are generated extracellular space.

Another pathological mechanism of AD is about tau protein. Closely related to the microtubule associated with the organization and structure of cells and the transport of intracellular substances, tau protein, as it is hyperphosphorylated by kinases such as GSK-3b, gets clustered and forms neurofibrillary tangles. Thus, Axoplasmic transport's function is not performed, resulting in brain cell death.

Although the exact cause of AD and the precise mechanism by which AD occurs remains unknown yet, many different hypotheses exist in terms of pathogenic mechanism: in addition to genetic mutations, the neurotransmitter hypothesis regarding decreased or increased levels of neurotransmitters, the amyloid hypothesis that cites nerve damage due to beta amyloid deposition as the cause of AD, the tau hypothesis about nerve damage due to hyperphosphorylation of tau protein, etc.

When it comes to AD's therapies currently used, cholinergic nervous system regulating drugs, such as donepezil (product name: Aricept), rivastigmine (product name: Exelon), and galantamine (product name: Razadyne), have a mechanism to improve the decreased function of transmitting signals. In the case of glutamate, as a neurotransmitter, causes the impairment of neurons, memantine (product name: Namenda) with NMDA receptor antagonistic mechanism inhibiting this is usually used. However, these drugs have the effect of just improving symptoms. Indeed, the drugs currently on the market are effective in alleviating symptoms such as cognitive impairment and memory loss and delaying the progress, but have various side effects. This is why there are limits in using them as therapeutic agents. Until the present, any disease modifying drug that can fundamentally treat AD has not existed, and those drugs currently on the market are capable of just easing symptoms and delaying the progress. Although a variety of therapies and medicines have been developed based on AD's pathological mechanism, these drugs for clinical use are not disease modifying ones and their effect is minor. Therefore, the development of a therapeutic drug for AD with a new-concept mechanism of action is urgently needed.

5.1.2. GV1001 and AD

GV1001, as a peptide (611–626, EARPALLTSRLRFIPK) composed of 16 amino acids derived from the active site of human telomerase reverse transcriptase (hTERT), was synthesized based on computer algorithms so that it is characterized by the powerful HLA class II binding. At the early stage of development, GV1001 was developed as an anticancer vaccine for the treatment of progressive pancreatic cancer, and then as its anticancer effect in various carcinomas, which was derived from the increase of immune reactions, was observed, its

efficacies and side effects have been evaluated through the clinical trials conducted for the treatment against cancers such as melanoma, non-small cell lung cancer, advanced hepatocellular carcinoma (Greten et. al., 2010), cutaneous T-cell lymphoma (Schlapbach et. al., 2011), and B-cell chronic lymphocytic leukemia (Kokhaei et. al., 2007). In nonclinical and clinical trials on GV1001, minor side effects such as flu-like symptoms and erythema were reported only at the administration site, and even in *in vivo* nonclinical trials, no definite side effects were reported. Particularly, according to the outcomes of clinical trials through the administration of GV1001, autoimmune side effects by hTERT-specific T cells, serious side effects, etc. have not been reported, securing safety in the body.

Considering that GV1001 is a material synthesized based on hTERT in addition to its excellent safety, it was judged that GV1001 could have the positive functions of the extra-telomeric effect, including cytoprotective, antioxidative and anti-inflammatory effects as well as the immunopotentiation activity of hTERT. In addition, several basic studies using *in vitro* and *in vivo* experiments confirmed that GV1001 has anti-inflammatory, anti-apoptotic, and anti-oxidant effects. Based on the hypothesis that it will have the effect in preventing and treating AD, therefore, the therapeutic efficacy of GV1001 for AD was evaluated in transgenic animal models having *in vitro* cell models and *in vivo* Alzheimer etiology; and by identifying the mechanism of action, the potential of GV1001, as a therapeutic agent for AD was proposed.

1) In order to identify the effect of GV1001 on the brain cell death due to neurotoxicity induced by amyloid-β, neural stem cells were separated from the rats' brain and then cultured. Thus, GV1001 was shown to effectively reduce the damage of neural stem cells induced by amyloid-β. In other words, this means that GV1001 is effective in preventing neurotoxicity induced by amyloid-β.

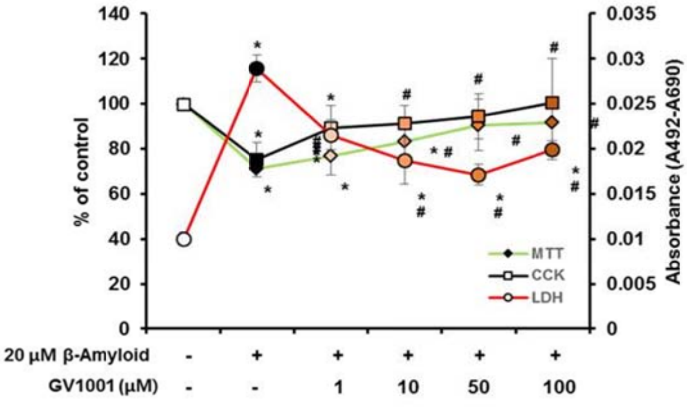

[Figure 1] Treatment of neural stem cell with GV1001 blocked the cell death, induced by Aβ fragment 25-35.

2) In order to check if GV1001 is capable of inhibiting apoptosis induced by amyloid-β in neural stem cells, the terminal deoxynucleotidyl transferase dUTP nick end labeling (TUNEL) was used to analyze the effect of GV1001 on apoptosis in neural stem cells, and as a result, GV1001 was shown to effectively prevent apoptosis induced by amyloid-β. This result suggests that it is capable of effectively blocking neurotoxicity induced by amyloid, in other words, neurotoxicity by amyloid-β plaque through actual apoptosis in Alzheimer patients.

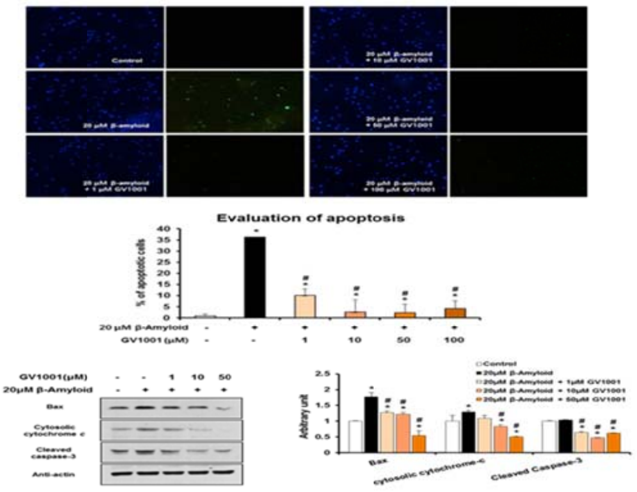

[Figure 2] GV1001 inhibited the apoptotic process caused by Aβ fragment 25-35.

3) In order to observe the effect of GV1001 on the inhibition of reactive oxygen species (ROS) generation induced by amyloid-β, neural stem cells were treated with amyloid-β fragment to determine an increase in ROS, and then a decrease in increased ROS due to GV1001 was observed. Therefore, GV1001 represents the protective effect for brain cells, by eliminating excess ROS generated and accumulated by amyloid-β, which become a big cause of the progress of AD.

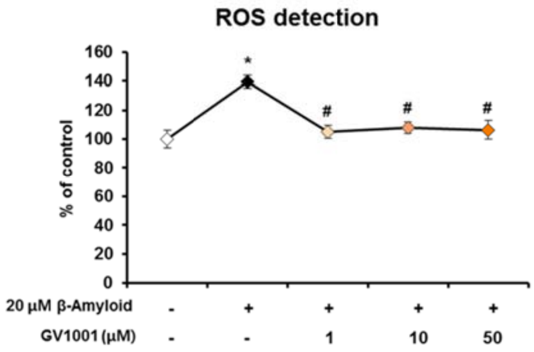

[Figure 3] GV1001 negatively affected the ROS production caused by Aβ fragment in neural stem cell.

4) Whether the decrease of the proliferative capacity of neural stem cells induced by treatment with amyloid-β could be effectively recovered by GV1001 was observed through the colony- forming assay using crystal violet staining. Although the proliferative capacity of neural stem cells was considerably decreased by amyloid-β fragments, treatment with GV1001 showed the proliferative capacity of neurons recovered again.

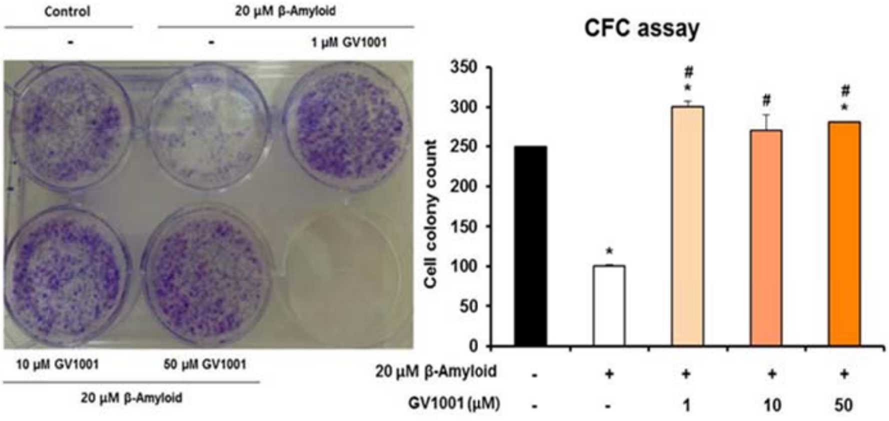

[Figure 4] GV1001 restored the proliferative property of neural stem cell.

5) In order to evaluate whether GV1001 showed the effect of improving cognitive function and memory ability in AD animal models, GV1001 was subcutaneously administered to AD mice called AD-3X Tg three times a week for two months. In the passive avoidance test, the administration of GV1001 recovered memory statistically significantly compared to the administration of saline. The test also confirmed that the longer latency led to the higher level of learning and memory.

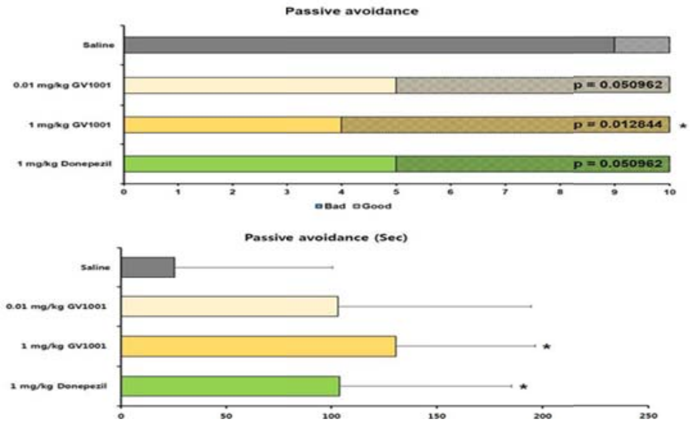

[Figure 5] GV1001 improved the cognitive and memory functions in AD mice.

6) After the passive avoidance test, the hippocampus was separated from the brain of transgenic mice with AD; the western blotting and immunofluorescence staining methods were performed to investigate and examine the effect of the administration of GV1001 on the variations in the amount of amyloid-β in the brain. The total amount of amyloid-β decreased by 84% in GV1001 (1mg/kg) group compared to saline group. The total amount of amyloid-β polysaccharide protein (oligomeric β amyloid) also decreased by 36% in GV1001 (1mg/kg) group compared to saline group.

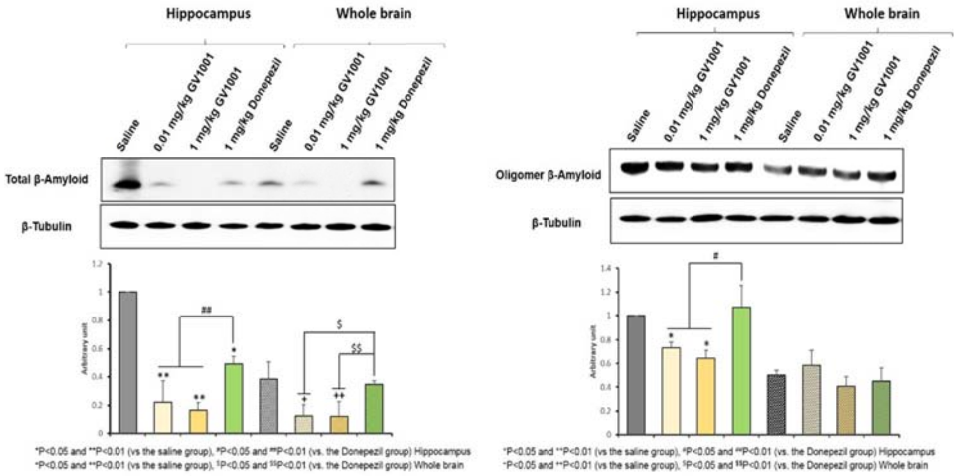

[Figure 6] GV1001 administration in AD-mice reduced the total β amyloid and oligomeric β amyloid level in both hippocampus and hemisphere

7) The western blotting and immunofluorescence staining methods were used on the brain separated from the test on AD animal models in order to observe the degree of neurofibrillary tangle formation. Tau protein considerably decreased by 35% in GV1001 (0.01 mg/kg) group compared to saline group, and decreased by 26% in GV1001 (1mg/kg) group compared to saline group. Both decrease rates were statistically significant.

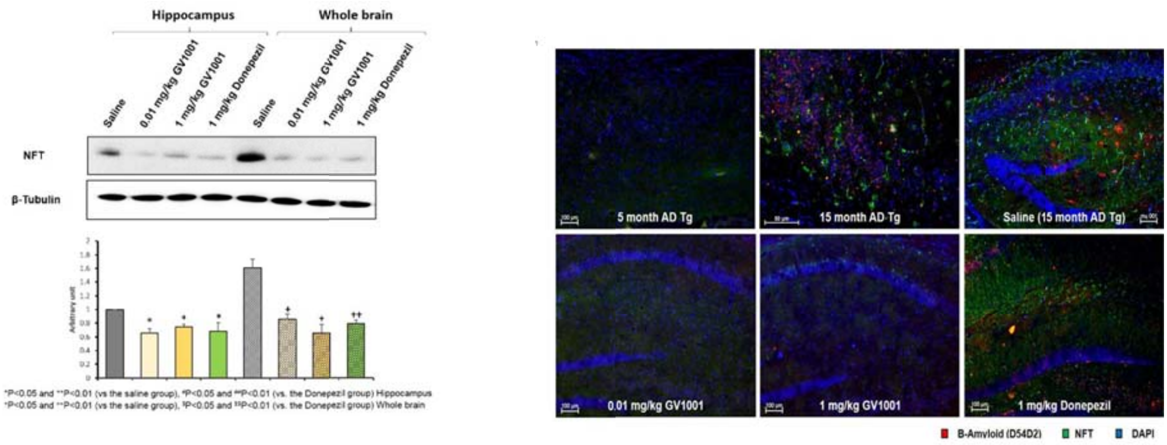

[Figure 7] GV1001 administration in AD-mice reduced the neurofibrillary tangles in both hippocampus and hemisphere.

8) In order to evaluate whether GV1001 could inhibit astrogliosis in AD animal models, the western blotting was performed using GFAP (Glial fibrillary acidic protein) antibodies to recognize activated astrocytes. The activation of astrocytes increased with aging and the development of AD statistically significantly decreased at least over 50% in both GV1001 (0.01, 1mg/kg) and donepezil (1mg/kg) groups.

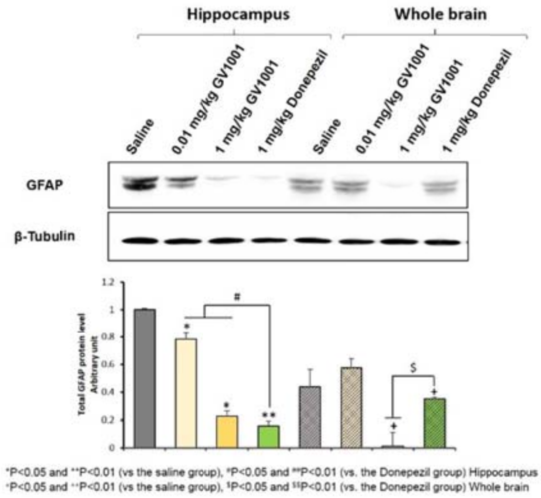

[Figure 8] GV1001 reduced astrocyte activation caused by Alzheimer pathology

9) In order to evaluate whether GV1001 was involved in neurogenesis or not, NeuN (mature neuron), DCX (immature neuron) and Tuj1(Beta tubulin III) were examined. NeuN (mature neuron), DCX (immature neuron) showed nearly statistically significantly doubled in

GV1001 (1mg/kg) group compared to saline group; and statistically significantly increased compared to positive control group donepezil. Likewise, it was also confirmed that a marker appearing in neural precursor cells and immature neurons were significantly increased by GV1001. This result suggests that GV1001 stimulated the voluntary neurogenesis occurring after brain damage and the activation of this neuron development process improved cognitive and memory functions.

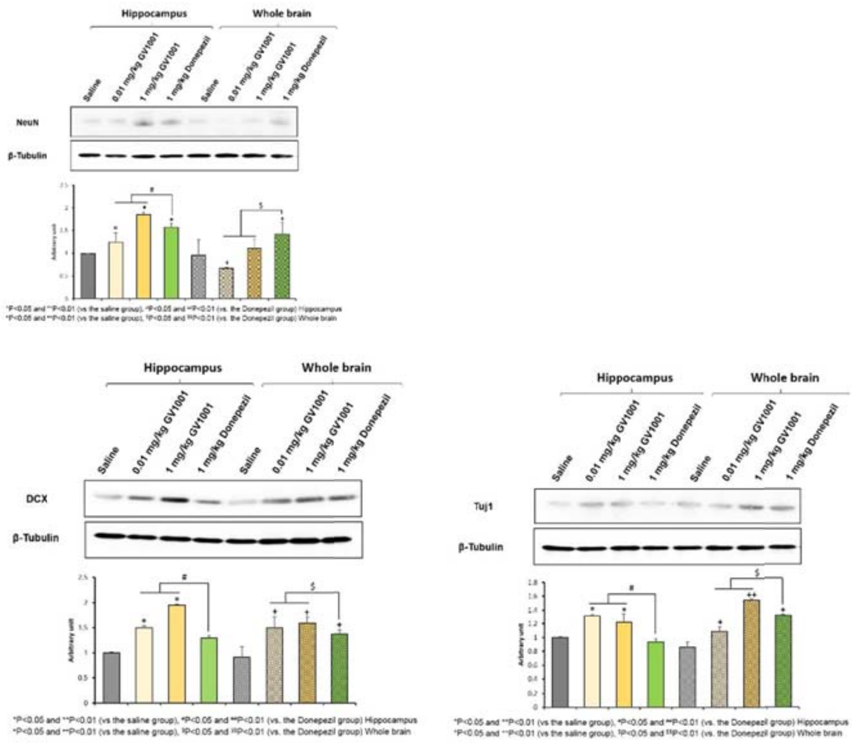

[Figure 9] GV1001 increased the neurogenesis in hippocampus and whole brains of AD mice.

5.1.3. GV1001's mechanism of action

GV1001 in AD animal models 1) considerably reduced the amount of amyloid-β, one of Alzheimer etiologies, 2) prevented the formation of neurofibrillary tangles due to the non-ideal transformation of tau proteins,and 3) inhibited astrogliosis due to the activation of astrocytes and simultaneously promoted neurogenesis. This *in vivo* result suggests that GV1001 used in Alzheimer patients can play a major role in recovery and rehabilitation of brain function, by adjusting the amount of amyloid-β, inhibiting the generation of a neurofibrillary tangle by tau, reducing the activation of brain cytotoxicity and astrocytes, and more activating neurogenesis by GV1001. Donepezil used for a positive control group in this study is a drug for dementia

used the most in the world. Nevertheless, GV1001 shows a comparative advantage over donepezil even in memory improvement and other mechanisms of action.

Although there have been ongoing detailed and excellent mechanism analysis studies at the molecular level, it is currently judged based on the accumulated results that GV1001 will be effective in treating AD by 1) decreasing the brain cell death induced by amyloid-β, 2) reducing the level of ROS generated by amyloid, and 3) through the mechanisms of action that prevent the impairment of neural stem cells' proliferative ability due to exposure to amyloid-β.

5.2. Necessity of Clinical Trial

This clinical trial was designed targeted at moderate to severe AD patients (K-MMSE≤19). Although AD has recently proliferated rapidly and subsequently socioeconomic costs have also increased dramatically, there are no special measures other than symptomatic therapy. The effects of drugs which have been considered to minutely improve symptoms are also minor, and various studies conducted around the world for the development of new drugs have a long way to go before producing performances. The main reason for this is indicated as a late treatment time. In other words, there has been a consensus that if patients already show symptoms, even any treatment may not be effective because brain lesions are already progressing considerably even though they are in the early stages of AD. Recently, a strong argument that the studies so far conducted toward only a single therapeutic target may be wrong has been raised. Therefore, various studies are being planned to develop not only a test method to diagnose AD more exactly and early but also a therapeutic method to treat several targets simultaneously. However, no successful results have been presented yet.

Through the previous studies which have been carried out so far, currently, this research staff were able to identify that when AD cell models and animal models were treated with GV1001, neuron death was inhibited and regeneration was stimulated through various mechanisms of action; thus the behavior function in animal models was improved and several pathologies by Alzheimer were also enhanced in post brain biopsies targeted at animal models, and further progressed lesions were reduced and improved. Taken together, it is considered very necessary to conduct a clinical trial to attempt a treatment fit for various disease stages using a therapeutic agent that can regulate various mechanisms involved in AD simultaneously, Therefore, it is judged that if this study can demonstrate that GV1001 has a sufficient potential as a fundamental therapeutic agent against AD and secures a mechanism of action and safety as a medicine, GV1001 will be able to become a fundamental drug with a completely different concept from other anti-AD drugs which have been applied so far for the actual clinical use and to completely replace existing therapeutic drugs.

In this clinical trial targeted at moderate to severe Alzheimer patients (K-MMSE ≤19), efficacy and safety among the three groups (placebo group, GV1001 0.56mg group, GV1001 1.12mg group) are compared and evaluated during the treatment Period (24 weeks). The primary efficacy variable of the study was the change from baseline in Severe Impairment Battery (SIB) score at Visit 15 (Week 24).

The secondary efficacy variables were the change from baseline in K-MMSE, Clinical Dementia Rating-Sum of Box (CDR-SOB), Neuropsychiatric Inventory (NPI), GDS, Alzheimer's Disease Cooperative Study-Activities of Daily Living (ADCS-ADL)-severe score, and the Clinician Interview-Based Impression of Change (CIBIC)-plus score at Visit 15 (Week 24). In addition, the safety and tolerability of GV1001 compared to placebo were assessed based on adverse events (AEs), clinical laboratory tests results, vital signs, and other observations related to safety (including electrocardiogram [ECG] recordings, physical examination and pregnancy test results). This clinical trial was designed to examine the efficacy and safety of GV1001 in AD patients.

6. OBJECTIVE OF CLINICAL TRIAL

The primary objective of this trial was to assess the efficacy and safety of GV1001 0.56 mg (Group 1) and GV1001 1.12 mg (Group 2) compared to Placebo, in the treatment of patients with a diagnosis of moderate to severe AD.

6.1. Primary Efficacy Endpoint

1) Change from baseline in the Severe Impairment Battery (SIB) score at Visit 15 (Week 24).

6.2. Secondary Efficacy Endpoint

1) Change from baseline in Korean Mini-Mental State Examination (K-MMSE) score at Visit 15 (Week 24).

2) Change from baseline in Clinical Dementia Rating-Sum of Box (CDR-SOB) score at Visit 15 (Week 24).

3) Change from baseline in Neuropsychiatric Inventory (NPI) score at Visit 15 (Week 24).

4) Change from baseline in Global Deterioration Scale (GDS) score at Visit 15 (Week 24).

5) Change from baseline in Alzheimer's Disease Cooperative Study-Activities of Daily Living-severe (ADCS-ADL-severe) score at Visit 15 (Week 24).

6) Change from baseline in Clinician Interview-Based Impression of Change-Plus (CIBIC-plus) score at Visit 15 (Week 24).

6.3. Safety Endpoint

1) Adverse events
2) Laboratory tests (Hematological tests, Blood Chemistry, Urinalysis)
3) Vital signs

7. INVESTIGATIONAL PRODUCTS USED IN CLINICAL TRIAL

7.1. Outline of Investigational Products

7.1.1. Investigational products (group1)

Investigational product	GV1001
Appearance	White freeze-dried powder in a colorless transparent glass vial
API/Dosage	Tertomotide hydrochloride (TertomotideHCl) 0.97 mg, 0.84 mg as Tertomotide
Excipient	No
Vial	3.5 mL disposable amount of a sterilized transparent vial (type I), sealed with a rubber stopper
Manufacturer	SAMSUNG PHARM Co., Ltd., Republic of Korea.
Supplier	GemVax & KAEL Co., Ltd., Republic of Korea.
Storage Temperature/Duration	• Kept into a sealed container at -25 ~ -15°C (Frozen) / until 60 months of the manufacture date
	• Kept into a sealed container at 2 ~ 8 °C (Refrigerated) / until 12 months of the manufacture date, and until 12 months after 43 months of frozen storage (-25 ~ -15°C)
Storage after Dilution	The solution should be administered as soon as possible within up to 6 hours after dilution, and kept refrigerated at 2-8°C until before administration.

7.1.2. Investigational products (group2)

Investigational product	GV1001
Appearance	White freeze-dried powder in a colorless transparent glass vial
API/Dosage	Tertomotide hydrochloride (Tertomotide HCl) 1.94 mg, 1.68 mg as Tertomotide
Excipient	No
Vial	3.5 mL disposable amount of a sterilized transparent vial (type I), sealed with a rubber stopper

Manufacturer	SAMSUNG PHARM Co., Ltd., Republic of Korea.
Supplier	GemVax & KAEL Co., Ltd., Republic of Korea..
Storage Temperature/Duration	• Kept into a sealed container at -25 ~ -15°C (Frozen) / until 60 months of the manufacture date<br>• Kept into a sealed container at 2 ~ 8 °C (Refrigerated) / until 12 months of the manufacture date, and until 12 months after 43 months of frozen storage (-25 ~ -15°C)
Storage of Dilution	The solution should be administered as soon as possible within up to 6 hours after dilution, and kept refrigerated at 2-8°C until before administration.

7.1.3. Investigational Reference Product(s)

0.9 % normal saline supplied by the study center.

7.2. Amount, Route and Duration of Administration

7.2.1. Amount and Route of Administration

1) Treatment Group 1 (GV1001 0.56mg)

0.30mL of 0.9% normal saline is put into **1** vial and gently stirred to dissolve it.

The concentration of Tertomotide in this prepared solution is 2.8mg/mL, and 0.2 mL of the solution is taken and subcutaneously administered.

The solution should be administered as soon as possible within up to 6 hours after dilution, and kept refrigerated at 2-8°C until before administration.

2) Treatment Group 2 (GV1001 1.12mg)

0.30mL of 0.9% normal saline is put into **1** vial and gently stirred to dissolve it.

The concentration of Tertomotide in this prepared solution is 5.6mg/mL, and 0.2 mL of the solution is taken and subcutaneously administered.

The solution should be administered as soon as possible within up to 6 hours after dilution, and kept refrigerated at 2-8°C until before administration.

3) Control Group (Placebo)

0.30mL of 0.9% normal saline is put into 1 vial and gently stirred, and 0.2 mL of the solution is taken and subcutaneously administered.

7.2.2. Duration of Administration

The investigational product and placebo are subcutaneously administered by SC injection once weekly for Visits 2 to 5 (Weeks 1 to 4) and once every 2 weeks for Visits 6 to 15 (Weeks 6 to 24).

7.3. Production, Packaging and Labeling of the Investigational Product

The investigational product is manufactured or purchased by the sponsor, and then supplied to the CRP of the study center.

As this clinical trial is conducted by the double-blind method, the formulation produced as a placebo of the investigational product should be the same in the appearance as each investigational product, should not be visually observed to be different from it and should not show a large difference in the weight. In addition, by attaching the same label, it should be ensured that the patient and the investigator are blinded. In order to do this, the manufacturer packs the vials filled with the investigational product and the investigational referance product into a plastic envelope so that the contents cannot be visible, and then the labeling is performed to notify about the blind damage of the investigation product and the code is generated to maintain blind.

The label of the investigational product is described in accordance with "Annex 11. Manufacturing of the Investigational Product of the Regulations on Good Manufacturing Practice." The description includes:

1. Indication of "For Clinical Trial"
2. Code name of product or generic name of main ingredient
3. Batch number or code number to identify the contents and packaging
4. The sponsor's name, address and phone number
5. Expiry date
6. Storage conditions
7. Reference code to identify clinical trial
8. If necessary, patient identification number, investigational product No., visit number

The investigational products will be subdivided and wrapped according to "7.2. Amount, Route and Duration of Administration" of this clinical trial protocol. Packaging of the investigational products to be used, pertinent to a total of 14 times per each patient from Visit 2 to Visit 15, should be prepared.

7.4. Management of Investigational Product

- The investigational product should be stored in a sealed container and be kept frozen (-25~-15℃), and not allowed to be used without the instructions (prescription) of the principal investigator or the subinvestigator.
- The sponsor should supply the investigational product, etc. to the CRP directly in consultation with the principal investigator, and should receive and preserve a receipt.
- The Clinical Trial Pharmacist should keep and manage the investigational product, etc. so that they are not used for any purpose other than clinical trial.
- The sponsor should check the quantity and storage conditions of the investigational products during this clinical trial and take appropriate measures to ensure that the clinical trial is properly conducted.
- In order to maintain the double blind, the non-blinded Clinical Trial Pharmacist for storing and managing the investigational product, and the non-blinded person in charge of administration for preparing and administering the investigational product are separately designated and managed by each institution.
- The non-blinded Clinical Trial Pharmacist, the non-blinded person in charge of administration, and the non-blinded monitor should ensure that both evaluators and patients are kept blinded by avoiding the tasks of disabling double blindness.
- In case of the discontinuation and termination of the clinical trial, the sponsor should collect and discard the unused investigational products. At this time, the Clinical Trial Pharmacist should return the non-dispensed investigational products to the sponsor and preserve a return receipt, after consultation with the principal investigator. The container (vial) of the investigational products, which are dispensed to patients and used during the clinical trial, should be discarded independently in accordance with the guidelines of each study center.

7.5. Maintenance of Blinding and Unblinding

To maintain double blind, in this clinical trial, the investigational product and the investigational reference product (placebo) will be packed so that no differences in the appearance are visibly observed between the two.

At the time when all patients have completed the trial, the unblinding can be done by performing the Database Locking. However, the information of the administration group, in case of unblinding, can be confirmed only by the sponsor and the CRO which conducts statistical analysis.

If the patient is required to be unblinded (the code is required to be opened) inevitably due to the occurrence of a serious ADR during the clinical trial; or this is deemed necessary by GemVax & KAEL Co., Ltd. for the patient's right and interest, and safety, only the unique code of the patient is allowed to be opened through the IWRS. In case unblinding is required, it can be made only for the patient by the investigator after consultation with the sponsor. The reason for unblinding and the date of unblinding should be documented and kept. The patient whose randomization code is unlocked cannot continue to participate in this clinical trial.

7.6. Concomitant Medications and Therapy/Prohibited Concomitant Medications and Therapy

7.6.1. Allowed concomitant medications

The following drugs are allowed to be administered concurrently over the duration of this clinical trial.

① The concomitant medications judged to have no effect on interpreting the results of this clinical trial among the medications taken by patients from before this clinical trial is allowed according to the investigator’s judgment.

② The medications used transiently for the purpose of treating other diseases or adverse events can be administered concurrently after discussing with a doctor in charge.

③ Donepezil taken at stable doses for 3 months or more prior to screening visit should be administered concurrently without changes in doses over the duration of this clinical trial.

Information about each drug (name of product, purpose of administration, dosage, duration of administration, etc.) in case of administration of all concomitant medications (including the therapeutic drugs for other diseases or adverse events) should be recorded in detail in the case report form (CRF).

7.6.2. Prohibited concomitant medications and therapy

The following concomitant medications are prohibited from screening (visit 1) to end-of-study visit.

Prohibited items are not limited to the concomitant medications and combination therapies specified below, and any medications judged by the investigator to have an effect on evaluating the efficacy of this clinical trial can also be prohibited.

If the prohibited concomitant medications need to be used for the treatment of patients over the duration of this clinical trial according to the judgment of investigators or doctors in charge

of other medical symptoms, the patients should immediately discontinue this clinical trial and this will have to be recorded in detail in the CRF.

① Other drugs for AD other than donepezil : Acetylcholinesterase inhibitors (rivastigmine, galantamine, etc.), NMDA receptor antagonists (memantine, etc.)
② Choline excitatory drugs (However, drugs for local administration such as choline agonists and pilocarpine collyrium are allowed.)
③ CNS stimulants (Methylphenidate, Modafinil, Pemoline, Atomoxetine)
④ Antidepressant drugs (tricyclic antidepressants, MAO inhibitors)
⑤ Typical antipsychotic drugs (Bromperidol, Chloropromazine, Haloperidol)
⑥ Anticholinergic drugs (Atropine, Glycopyrrolate, Scopolamine, Homatropine, Ipratropium) However, the short-term use for the purpose of alleviating the symptoms of digestive system is allowed.)
⑦ All investigational products or medical devices not specified in this trial.
⑧ Use of any abused drug (including but not limited to illegal amphetamines, hemp, cocaine, illegal opiate drugs, propoxyphene, methadone, methaqualone, phencyclidine or illegal barbiturates)
⑨ Previous administration of all investigational vaccines for AD
⑩ Regular use of narcotics (for 3 days or more a week)
⑪ Other drugs affecting the immune system

7.6.3. Drugs requiring caution

The following drugs can be taken over the duration of this clinical trial, but careful administration is required. The drug name, administration & dosage, and administration period should be recorded in the CRF.

① Anticholinergic drugs (antispasmodics, asthma drugs, etc. which represent anticholinergic effect except prohibited medications)
② Sleeping pills (fast-acting sleeping pills ; drugs known to affect the central nervous system such as zolpidem, brotizolam, lormetazepam, rilmazafone, zopiclone, ramelteon), anticonvulsants, atypical antipsychotics (seroquel, cacepin, etc.), anxiolytics, antidepressants (tricyclic antidepressants, antidepressants other than MAO inhibitors, SSRIs, etc.)
③ In case of drugs that can affect the efficacy evaluation of the investigational product such as Vitamin E, Ginko biloba extracts, estrogens, and brain function improvers (citicoline, oxiracetam, piracetam, acetyl-L-carnitine, Nimodipine, etc.), the administration of them is acceptable without changes in dosage and administration over the duration of this clinical

trial as long as they are administered at intermittent, low doses for a short period of time (≤ 2 months in a row) and at stable doses from 2 weeks prior to the administration of the investigational product. However, the cognitive function should be evaluated 3 hours after the time when a drug that affects the efficacy evaluation of the investigational product is taken.

8. TARGET DISEASE

Moderate to severe Alzheimer's disease

9. INCLUSION CRITERIA, EXCLUSION CRITERIA, TARGET SAMPLE SIZE AND JUSTIFICATION

9.1. Inclusion Criteria

Patients must have met all the following criteria for inclusion in the study:

1) Aged ≥ 55 to ≤ 85 years, inclusive, at the time of signing the informed consent.
2) Meet the Diagnostic and Statistical Manual of Mental Disorders, fourth edition (DSM-IV) criteria for diagnosing dementia.
3) Clinically diagnosed with probable AD as defined in the National Institute of Neurological and Communicative Disorders and Stroke (NINCDS) and the Alzheimer’s Disease and Related Disorders Association (ADRDA) criteria.
4) Korean Mini-Mental Status Examination score ≤ 19 at the screening visit.
5) Rated as Grade 5 to 6 on the GDS.
6) Have no other diseases to cause dementias other than AD as a result of an MRI or CT scan within 12 months prior to the screening visit.
7) Received donepezil at stable doses for 3 months or more prior to the screening visit.
8) Able to undergo cognitive and other tests by walking on their own or visiting hospitals using an assist device on an outpatient basis or for hospitalization.
9) Have a guardian who is able to accompany the patient for all visits, supervise the patient's compliance with the procedures specified in the clinical trial protocol and the investigational product, and provide detailed patient information.
10) Voluntarily agreed to participate in this clinical trial and signed the patient consent form (if there is no legally authorized representative, the role should be assumed in the order of a spouse, a lineal ascendant, and a direct descendant. If there are several lineal ascendants or direct descendants, consultation is needed, and if no agreement is reached, the oldest should become a legally authorized representative*.)

* Paragraph 2, Article 16 of the Act on Bioethics and Safety.

9.2. Exclusion Criteria

Patients who met any of the following exclusion criteria were not included in the study:

1) gnosis of other causes of dementia as listed below as a result of a CT/MRI test and neurologic examination within 12 months prior to screening or at the screening visit:
   - Possible, probable or definite vascular dementia according to the National Institute of Neurological Disorders and Stroke and the Association Internationale pour la Recherche et l'Enseignement en Neurosciences (NINDS AIREN) criteria.
   - Other central nervous system diseases that may cause the impairment of cognitivefunction(cerebrovascular disease including cerebrovascular dementia, Parkinson's disease, Huntington's disease, subdural hematoma, normal pressure hydrocephalus, brain tumor, Creutzfeldt-Jakob disease).
   - Neuropathy such as delusion, delirium, epilepsy.
2) Abnormal laboratory test results which are considered to contribute to the severity of their dementia or are a cause of dementia e.g. vitamin B12/folic acid levels, abnormal syphilis serology, and thyroid stimulating hormone (TSH) levels.
3) History of depression or a history of significant psychiatric illness which according to the investigator's judgment may interfere with the participation of this clinical trial, such as schizophrenia or bipolar affective disorders.
4) History of known or suspected seizures, including febrile seizure, or recent unexplained loss of consciousness, or a history of significant head trauma accompanied by loss of consciousness.
5) Acute or unstable cardiovascular disease, active peptic ulcer, or uncontrolled hypertension, uncontrolled diabetes or insulin dependent patients, or any medical condition that may interfere with the completion of the clinical study.
6) History of hypersensitivity to GV1001 or to medicinal products with similar chemical structures.
7) History of alcohol and drug abuse or dependence (except nicotine dependence) within the last 2 years.
8) History of cancer within the past 5 years, except non-metastatic skin basal cell carcinoma and/or skin squamous cell carcinoma, carcinoma in situ of uterine cervix, or non-progressive prostate cancer.
9) Renal dysfunction (define as creatinine clearance [CLcr] < 30 mL/min).
10) Serious hepatic dysfunction (define as ALT or AST≥ 2.0 times the upper limit of normal).

11) Administered drugs other than donepezil to treat AD or other cognitive function impairments.
12) Prohibited medications and therapies specified in the Clinical Protocol, Section 7.6.2, such as anticholinergic drugs, choline excitatory drugs antidepressant drugs (tricyclic antidepressants, monoamine oxidase inhibitors), typical antipsychotic drugs, central stimulant, and other drugs for AD other than donepezil or who are scheduled to receive them over the duration of this clinical trial.

Note: Drugs for local administration such as pilocarpine collyrium are permitted.

13) Women of reproductive age without a negative pregnancy test and without a commitment to using an acceptable method of contraception (e.g., condoms, diaphragms, oral contraceptives and long acting progestin agents), if sexually active, until the end of the trial. Women who are postmenopausal (1 year since last their menstrual cycle), surgically sterilised or who have undergone a hysterectomy are considered not to be reproductive and can be included.
14) Pregnant or lactating women.
15) Participated in a clinical study with any experimental treatment within 4 weeks prior to the screening visit or previous participation in the present study.
16) Weight ≤ 35 kg at screening and admission visits.
17) Previous treatment with the investigational product.
18) Participated in Alzheimer's-type dementia vaccine clinical trial (e.g.: amyloid vaccine) within 6 months prior to the clinical trial.
19) Any other condition that in the opinion of the investigator can interfered with the interpretation of the study results or constituted a health risk for the patient if he/she take part in the study.

9.3. Justification for Sample Size Calculation

	Treatment group 1	Treatment group 2	Control group	Total sample size
Sample size for efficacy evaluation	24	24	24	72
Sample size including dropout rate(20%)	30	30	30	90

This clinical study is designed to evaluate the ability of GV1001 to reduce disease progression and to assess its safety profile in moderate to severe AD patients. The study planned to enroll at a total of 90 patients (sample size eligible for efficacy evaluation: 72). The sample size for this study to evaluate efficacy was not determined based on statistical considerations because GV1001 was different in mechanism of action and effectiveness from other existing

medications. However, the sample size is calculated by examining and comparing the domestic and overseas literature reviews of multicenter, randomized, double-blind, placebo-controlled, parallel design, and prospective phase II clinical studies, in addition to historical clinical and experimental animal data.

10. STUDY PERIOD

24 months from the IRB approval date

11. STUDY METHOD

11.1. Design

This clinical trial is a pilot study for therapeutic exploration to regulate severity of disease, control progress of disease, alleviate symptoms, and set monotherapy or additive therapy of donepezil when the GV1001 manufactured by GemVax & KAEL Co., Ltd. is subcutaneously administered to moderate to severe Alzheimer patients at a dose of 0.56 or 1.12 mg/day. In order to evaluate the efficacy and safety of GV1001 between GV1001 0.56mg group and GV1001 1.12mg group, this clinical trial was designed as a multi-center, randomized, double-blind, placebo-controlled, parallel design, prospective phase II clinical trial.

If a patient or a legally authorized representative agrees in writing to participate in this clinical trial at the screening visit, the necessary checkup and examination will be carried out during the screening visit in accordance with the clinical trial protocol and the screening lasts within 2 weeks.

For patients considered fit for the inclusion criteria as a result of evaluating the suitability as patients of this trial after the completion of screening, they are assigned to treatment group 1 (GV1001 0.56 mg), treatment group 2 (GV1001 1.12 mg), or control group (placebo) in the ratio of 1 to 1 to 1; and are treated with the investigational product or placebo 4 times at intervals of 1 week and then 10 times at intervals of 2 weeks (a total of 14 times) to evaluate their safety and efficacy.

In order to ensure the objectivity and accuracy of trial results, efficacy evaluators should be limited to those who have sufficiently received education and training, and efficacy evaluation per visit should be conducted in a fixed order.

Donepezil, which has been stably administered for 3 months or more before screening, can be administered without changes in dosage over the duration of clinical trial.

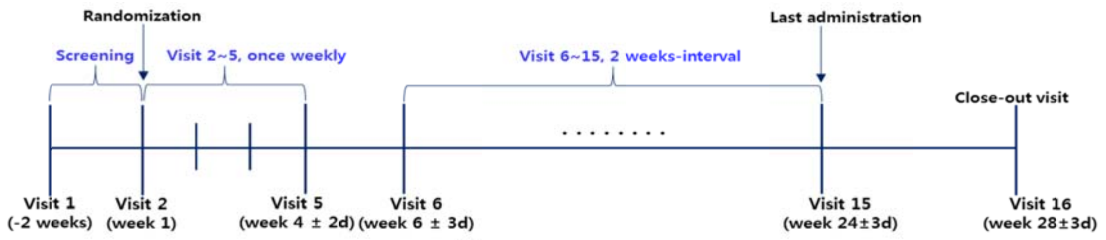

[Study Flow]

11.2. Assignment to Administration Groups

The patients who agree in writing to participate in this clinical trial will receive the screening number composed of [institution number 2 digits]-S-[serial number 3 digits] in the order that the written consent is made. After that, patients who meet all of the inclusion criteria and none of the exclusion criteria will be assigned randomly according to the randomization plan, and receive a unique random assignment number consisting of the R-[serial number 3 digits] in the order that the investigator determines the enrollment of patients.

The patients who meet all of the inclusion criteria and none of the exclusion criteria will be assigned to each administration group (investigational product code, random number), together with an applicable investigational product, through the Interactive Web Response System (IWRS).

The ratio of assignment per group will be 1 to 1 to 1 in this clinical trial. The randomization list will be created by statisticians via SAS v.9.4 or higher version program according to the predetermined bundle unit, and the size of the bundle unit will be set to a predetermined size but will not disclosed in this protocol.

GemVax & KAEL Co., Ltd. will attach the label of investigational product to its package, and then transfer the investigational product to a pharmacist in the relevant clinical trial institution.

12. OBSERVATION ITEMS, CLINICAL EXAMINATION ITEMS AND OBSERVATIONAL EXAMINATION METHODS

12.1. Evaluation Method of Clinical Trial

12.1.1. Efficacy evaluation

In order to ensure the objectivity and accuracy of trial results, efficacy evaluators should be limited to those who have sufficiently received education and training, and efficacy evaluation per visit should be conducted in a certain order.

12.1.1.1. SIB (Severe Impairment Battery)

SIB evaluation will be conducted at Visits 2, 9 and 15.

The SIB (Saxton et al., 1990) was developed to evaluate dementia patients with severe cognitive impairment and consists of easy and simple questions. The questionnaire consists of 50 questions for the 9 items of “orientation”, “social interaction”, “construction”, “orienting to name”, “language”, “memory”, “praxis”, “visuo-spatial ability”. The scores range from 0 to 100 points, and the better the state of the patient, the higher the score.

12.1.1.2. K-MMSE (Korea-Mini-Mental State Examination)

K-MMSE evaluation will be conducted at Visits 1, 9 and 15.

The Mini-Mental State Examination (MMSE), is a screening test tool to measure cognitive status (Folstein et al., 1975). In Korea, the MMSE was translated into 2 versions, the Korean version of the Mini-Mental State Examination (MMSE-K) (Park and Kwon, 1990) and the Korean Mini-Mental State Examination (K-MMSE) (Kang et al., 1997). This trial used the K-MMSE which consists of "orientation", "registration", "attention and calculation", "recall", and "linguistic and visuo-constructive skills". The scores range from 0 to 30 points, and the higher the level of cognitive impairment, the lower the score.

12.1.1.3. CDR-SOB (Clinical Dementia Rating)

CDR-SOB evaluation will be conducted at Visits 2,9 and 15.

The CDR is a global assessment instrument that yields global and Sum of Boxes (SOB) scores (Morris, 1993; Hughes et al., 1982). It is obtained through semi-structured interviews of patients and caregivers, and cognitive functioning is rated in 6 domains of functioning: “memory”, “orientation”, “judgment and problem solving”, “community affairs”, “home and hobbies”, and “personal care”. Each domain is rated on a 5-point scale of functioning as follows: 0, no impairment; 0.5, questionable impairment; 1, mild impairment; 2, moderate impairment; and 3, severe impairment (personal care is scored on a 4-point scale without a 0.5 rating available). The CDR-SOB score is obtained by summing each of the domain box scores; the total score is on the basis of 30 points.

12.1.1.4. CIBIC-plus (Clinician Interview-Based Impression of Change-Plus)

CIBIC-Plus evaluation will be conducted at Visits 2, 9 and 15.

The CIBIC-plus is a tool to evaluate the general functional status of dementia patients. It consists of 4 items: "general conditions", "mental and cognitive skills", "activity", and "daily function"; and is conducted in the form of semi-structured interview. A Clinician Interview-Based Impression of severity (CIBIS) 7 point scale* was carried out to assess severity as a baseline, and CIBIC-plus score** was carried out to evaluate the degree of changes in general function status on a scale of 1 to 7, referring to the results of CIBIS as a baseline, at follow-up visits. The evaluation is not related to any aspect of patients' treatment and evaluation and is performed by independent testers who cannot see data of other patients participating in this clinical trial.

*CIBIS 7-point scale

1 : Normal, not at all ill
2 : Borderline mentally ill
3 : Mildly ill
4 : Moderately ill
5 : Markedly ill
6 : Severely ill
7 : Among the most extremely ill patients

**CIBIC-plus score

1 : Very Much Improved
2: Much Improved
3: Minimally Improved
4: No change
5: Minimal worsening
6 : Moderate worsening
7 : Marked worsening

12.1.1.5. NPI (Neuropsychiatric Inventory)

NPI evaluation will be conducted at Visits 2, 9 and 15.

The NPI evaluation was developed to assess behavior disorders in dementia patients (Cummings et al., 1994; Cummings, 1997). The NPI measures the frequency and severity of behavior disorders. It is composed of 12 areas: “apathy”, “depression”, “agitation/aggression”, “anxiety”, “sleep disorder”, “irritability”, “appetite disorder”, “aberrant motor behavior”, “delusion”, “disinhibition”, “hallucination”, and “euphoria”, and each area includes selected questions and detailed questions. The detailed questions consist of the frequency of symptoms on a 4-point scale and the severity of symptoms on a 3-point scale. The score in each area is achieved by multiplying the frequency score of symptoms in each area by the severity score of symptoms; the total score of NPI is calculated as an aggregate of the scores for each area.

12.1.1.6. GDS (Global Deterioration Scale)

GDS evaluation will be conducted at Visits 1, 9 and 15.

The GDS is a tool to evaluate the clinical features and severity of dementia in 7 stages (Reisberg et al., 1982). It is useful in assessing the progress or prognosis of treatment because it can detect stage changes over time. Evaluation is based on the total score of all questions, and changes in scores before and after the administration of the Investigational product and the significance of differences between Investigational product group and the Placebo group are evaluated.

12.1.1.7. ADCS-ADL-severe (Alzheimer’s Disease Cooperative Study-Activities of Daily Living scale-severe)

ADCS-ADL-severe will be conducted at Visits 2, 9 and 15.

The ADCS-ADL-severe tool is used to evaluate daily living activity in moderate to severe dementia patients (Galasko et al., 1997). The ADCS-ADL tool consists of 45 items with 19 questions including "Have you washed your hands or face without help for the last 4 weeks?", "Have you turned off the tap water after using it without help?" "Have you turned on the light for yourself when entering a dark room?" and "Have you turned off the light for yourself when going out a room or going to sleep?".

12.1.2. Safety evaluation

1) Adverse events

Investigators are trained to voluntarily report information on adverse events to patients frequently, and should check if there are adverse events through interviews, questionnaires,

etc. during regular or additional visits. The examination includes onset date, disappearance date, severity and results of adverse events, measures taken related to investigational product / investigational reference product, causal relationship with investigational product/control drug, and treatment status of adverse events and the details.

2) Laboratory tests

The laboratory tests will be conducted on all patients at Visits 1, 9 and 15 to assess their systemic health status. All of the inclusion criteria and exclusion criteria, including clinical laboratory test results, must be checked before the administration of the investigational product. Clinically significant abnormal results at the time of screening will be recorded as a medical history, and subsequent clinically significant abnormal results will be recorded as an adverse event.

In addition to evaluations according to the schedule, additional tests are possible if the investigator judge as necessary.

In case of a visit scheduled for blood sampling prior to clinical laboratory tests, fasting (drink and food prohibition except water) is required to patients. As for the screening (Visit 1) test, if there are results of a test conducted within 4 weeks, they can be used. In addition, reexamination can be carried out according to the investigator's judgment. Test items include:

- Hematological tests : WBC, RBC, Hemoglobin, Hematocrit, Platelets count, WBC Diffcount(Neutrophils, Lymphocytes, Monocytes, Eosinophils, Basophils)
- Blood Chemistry : BUN, Creatinine, Uric acid, Total bilirubin, Albumin, Total Protein, ALT, AST, γ-GTP, Alkaline phosphatase, Glucose, Total Cholesterol *Only during screening visits: Vitamin B12, Folic acid, HbA1c
- Urinalysis : Protein(Albumin), Glucose, Ketones, WBC, Blood(RBC)
- Blood coagulation test : INR (Only during screening visits)
- Thyroid function tests : TSH, free T4 (Only during screening visits)
- Syphilis, AIDS tests : VDRL, anti-HIV (Only during screening visits)

Serum creatinine clearance is determined at the time of screening using the following Cockcroft-Gault formula.

$$CL_{cr} = \frac{[(140 - Age\ (years)] * Weight\ (kg) * 0.85(for\ female\ patients)}{72*Serum\ Creatinine\ (mg/dl)}$$

3) Pregnancy test

For women of childbearing age except patients (including surgical menopause) who have been confirmed to undergo a sterilization operation (hysterectomy, bilateral oophorectomy, bilateral tubal ligation, etc.) and reach menopause (in case the continuous amenorrhea lasts 12 months or more), the state of pregnancy will be checked through the urine hCG test at Visits 1 and 15.

4) Measurements of vital signs, height and body weight

Vital signs should be monitored prior to the tests scheduled for each visit according to the clinical trial flow chart. After taking a rest for 5 minutes, the systolic / diastolic blood pressure, pulse rate, respiration and body temperature should be measured in a sitting position.

Height should be measured only at Visit 1. When body weight and height are measured, patients should take off outer clothing and shoes and empty their pockets. The body weight scale should be always the same if possible whenever used at each visit.

5) Physical examination

Physical examinations should be conducted at each visit according to the clinical trial flow chart and include checkups of outer appearance, skin, head/neck, chest/lung, heart, abdomen, urinary system/reproductive system, limbs, musculoskeletal system, nervous system, and lymph node.

Clinically significant findings detected through physical examinations before initiating the administration of the investigational product will be collected as a medical history; and if significant physical examination findings consistent with the definition of adverse events are detected after initiating the administration of the investigational product, they will be collected as an adverse event.

6) ECG

A 12-Lead Electrocardiography (ECG) should be conducted at Visit 1. Clinically significant abnormal results will be recorded as a medical history. As for the screening (Visit 1) test, if there are results of a test conducted within 3 weeks, they can be used. In addition, reexamination can be carried out according to the investigator's judgment.

12.1.3. Others

1) Basic information on patients

Prior to initiating this clinical trial, the objectives and contents of this clinical trial should be explained in detail to patients. After the signed informed consent was completed, the screening number will be given to each patient in the order of consent, and then basic information of patients will be investigated.

Matters of record include whether written consent was completed, date of consent, gender, date of birth, age, etc.

2) Investigation of medical history and medication history

Each patient's medical history and medication history will be investigated and recorded in detail through interview and previous medical records. Investigation of medical history and medication history should include the following contents:

Regarding the medical history, past medical history including surgical history and present illness within 6 months before participation in this clinical trial will be investigated; and status of hypersensitivity, time of onset (year, or year and month of onset), investigator's opinion, etc. will also be recorded.

As for medication history, the investigators will examine the name of drugs administered within 4 weeks at the time of screening (Visit 1) and administration status (administration & dosage, period and purpose of administration).

The investigators should also check if there are any changes in comparison to the matters identified in the investigation of medication history at the time of screening, and record any changes in the concomitant medication in detail in the CRF.

12.2. Per-visit Procedures

12.2.1. Visit 1 (within -2 weeks; screening)

The patients who are selected to participate in this clinical trial will be informed of this trial and evaluated in the following order.

1) Prior to initiating this clinical trial, the entire processes of this clinical trial will be explained to patients or legally acceptable representatives, and the written informed consent will be completed.
2) The screening number will be given to each patient in the order of consent
3) Each patient's demographic information, medical history (past medical history including surgical history and present illness), and medication history will be investigated/recorded.
4) Physical examination will be carried out.
5) Vital signs (blood pressure, pulse rate, respiration, and body temperature), height, and body weight will be measured.
6) ECG will be conducted.
7) Laboratory tests will be conducted (Tests of blood coagulation, thyroid, syphilis, AIDS, Serum Creatinine Clearance, vitamin B12, folic acid, and HbA1c will be carried out only during screening visits).
8) For female patients of childbearing age, pregnancy test will be conducted.
9) K-MMSE test will be conducted.
10) GDS test will be conducted.
11) Inclusion/exclusion criteria will be checked.
12) The next visit date (Visit 2) will be set.
13) Previous medication (medication previously administered)/concomitant medication will be checked.
14) Whether there is another cause of dementia will be checked through CT and MRI brain scans. However, if there are results of CT and MRI conducted within 12 months of screening, they can be replaced.

12.2.2. Visit 2 (1 week: Randomization and Administration of the Investigational Product)

This visit will be conducted within 2 weeks after Visit 1, and if there are tests not performed at Visit 1, they will be checked over the duration of screening. Evaluations to be made at this visit are as follows:

1) Any change in medical history and concomitant medication will be checked in comparison to last visit.
2) Physical examination will be carried out.

3) Vital signs (blood pressure, pulse rate, respiration, and body temperature), height, and body weight will be measured.
4) Final assessment will be made with regard to whether the patients meeting the inclusion/exclusion criteria have been selected by putting together the results of all the tests and evaluations including clinical laboratory tests.
5) Randomization will be conducted.
6) SIB test will be conducted.
7) CDR-SOB test will be conducted.
8) CIBIC-Plus test will be conducted.
9) NPI test will be conducted.
10) ADCS-ADL-severe test will be conducted.
11) The investigational product will be administered (Whether there is any AE will be observed for 30 minutes after the administration).
12) The next visit date (Visit 3) will be set.

12.2.3. Visits 3~5 (2, 3, and 4 weeks ± 2 days ; Administration of the Investigational Product)

This visit will be conducted at intervals of 1 week after Visit 2, and evaluations to be made at this visit are as follows:

1) Any change in concomitant medication will be checked in comparison to last visit.
2) Physical examination will be carried out.
3) Vital signs (blood pressure, pulse rate, respiration, and body temperature), height, and body weight will be measured.
4) Incidence of AEs and the details will be investigated.
5) The investigational product will be administered (Whether there is any AE will be observed for 30 minutes after the administration).
6) The next visit dates (Visits 4, 5, and 6) will be set.

12.2.4. Visits 6~8 (6, 8, and 10 weeks ± 3 days ; Administration of the Investigational Product)

This visit will be conducted at intervals of 2 weeks after Visit 5, and evaluations to be made at this visit are as follows:

1) Any change in concomitant medication will be checked in comparison to last visit.
2) Physical examination will be carried out.

3) Vital signs (blood pressure, pulse rate, respiration, and body temperature), height, and body weight will be measured.
4) Incidence of AEs and the details will be investigated.
5) The investigational product will be administered (Whether there is any AE will be observed for 30 minutes after the administration).
6) The next visit dates (Visits 7, 8, and 9) will be set.

12.2.5. Visit 9 (12 weeks ± 3 days ; Administration of the Investigational Product)

This visit will be conducted at 12 weeks after Visit 1, and evaluations to be made at this visit are as follows:

1) Any change in concomitant medication will be checked in comparison to last visit.
2) Physical examination will be carried out.
3) Vital signs (blood pressure, pulse rate, respiration, and body temperature), height, and body weight will be measured.
4) Laboratory tests will be conducted.
5) SIB test will be conducted.
6) K-MMSE test will be conducted.
7) CDR-SOB test will be conducted.
8) CIBIC-Plus test will be conducted.
9) NPI test will be conducted.
10) GDS test will be conducted.
11) ADCS-ADL-severe test will be conducted.
12) Incidence of AEs and the details will be investigated.
13) The investigational product will be administered (Whether there is any AE will be observed for 30 minutes after the administration).
14) The next visit date (Visit 10) will be set.

12.2.6. Visits 10~14 (14, 16, 18, 20, and 22 weeks ± 3 days ; Administration of the Investigational Product)

This visit will be conducted at intervals of 2 weeks after Visit 9, and evaluations to be made at this visit are as follows:

1) Any change in concomitant medication will be checked in comparison to last visit.
2) Physical examination will be carried out.

3) Vital signs (blood pressure, pulse rate, respiration, and body temperature), height, and body weight will be measured.
4) Incidence of AEs and the details will be investigated.
5) The investigational product will be administered (Whether there is any AE will be observed for 30 minutes after the administration).
6) The next visit dates (Visits 11, 12, 13, 14, and 15) will be set.

12.2.7. Visit 15 (24 weeks ± 3 days ; Administration of the Investigational Product)

This visit will be conducted at 24 weeks after Visit 1, and evaluations to be made at this visit are as follows. Even the patients who drop out of this clinical trial should undergo the tests conducted at Visit 15.

1) Any change in concomitant medication will be checked in comparison to last visit.
2) Physical examination will be carried out.
3) Vital signs (blood pressure, pulse rate, respiration, and body temperature), height, and body weight will be measured.
4) Laboratory tests will be conducted.
5) SIB test will be conducted.
6) K-MMSE test will be conducted.
7) CDR-SOB test will be conducted.
8) CIBIC-Plus test will be conducted.
9) NPI test will be conducted.
10) GDS test will be conducted.
11) ADCS-ADL-severe test will be conducted.
12) Incidence of AEs and the details will be investigated.
13) The investigational product will be administered (Whether there is any AE will be observed for 30 minutes after the administration).
14) For female patients of childbearing age, pregnancy test will be conducted.
15) The next visit date (Visit 16) will be set.

12.2.8. Visit 16 (28 weeks ± 3 days; End of Trial)

This visit will be conducted at 28 weeks ± 3 days after Visit 1.

1) Any change in concomitant medication will be checked in comparison to last visit.
2) Physical examination will be carried out.

3) Vital signs (blood pressure, pulse rate, respiration, and body temperature), height, and body weight will be measured.
4) Incidence of AEs and the details will be investigated.

12.2.9. Additional visits

Additional visits may be made frequently in addition to scheduled visits, if judged as necessary by the request of the patient or the patient's representative, or at the discretion of the investigator, and necessary examinations will be conducted according to the investigator’s judgment.

13. PREDICTED ADVERSE EVENTS AND PRECAUTIONS

The safety and tolerability of the investigational product GV1001 used in this clinical trial were confirmed through previous trials. The most frequent adverse events included gastrointestinal diseases, such as nausea, vomiting, diarrhoea, abdominal pain, and fever. Serious adverse events (SAEs) reported in patients treated with GV1001 (alone or in combination with other drugs) in all GV1001 clinical trials totaled 1,418, and most SAEs (901 out of 1,418 cases) were reported in TeloVac Study.

SAEs reported most frequently in all GV1001 clinical trials are as shown in the table below:

Table 1. SAEs of GV1001 Incurred in Previous Trials

Gastrointestinal disorders	409/1,418 cases (28.60%)
Abdominal pain	80
Subileus	54
Ascites	45
Nausea	44
Constipation	27
Diarrhoea	25
Obstruction gastiric	15
Vomiting	14
Duodenal obstruction	12
Etc.	93
General disorders and administration site conditions	280/1,418 cases (19.58%)

Disease progression	108
Pyrexia	65
Device occlusion	29
Pain	15
General physical health deterioration	13
Chills	8
Fatigue	8
Etc.	49
Infections and infestations	196/1,418 cases (13.71%)
Biliary sepsis	39
Sepsis	22
Infection	20
Lower respiratory tract infection	17
Pneumonia	15
Cellulitis	10
Urinary tract infection	7
Liver abscess	5
Etc.	61

In addition to SAEs reported frequently, erythema, soreness, pain, etc. at the injection site were reported. The injection site reactions are the side effects occurring frequently when GV1001 is administered. Generally, the reactions occur within 24 hours after the administration of GV1001 and disappear within days. Therefore, the above adverse events may occur even in this clinical trial.

If immunity-related adverse events specific to this drug appear, the administration of this drug should be discontinued, and treatments of symptoms or supportive treatments should be carried out according to the investigator's judgment while the immune response is decreasing.

14. CRITERIA OF TRIAL DISCONTINUATION AND DROPOUTS

14.1. Criteria of Trial Discontinuation and Dropouts

Patients are free to withdraw from this clinical trial at their request at any time. The investigator or sponsor is also free to withdraw a patient at any time for the reasons of safety, action, and administration.
The investigator should inquire of the patient about a cause of dropout in this trial and ask him/her to make the last visit so that all tests can be performed as scheduled at Visit 15. If the patient does not make the scheduled visit, the investigator should make every effort to contact him/her and document results of tests related to the patient as best he/she can. The investigator should also do his/her best to identify the reason why the patient dropped out of this trial, ask for the return of unused investigational products, and continue to monitor the patient (follow-up) with regard to unresolved adverse events.

In case the patient withdraws his/her consent, further evaluation and additional data collection will no longer be possible, even if agreement to the personal information disclosure is withdrawn. All data collected prior to the withdrawal of consent can be continuously kept and used by the sponsor.

The following are the cases where the patient can stop and drop out of this clinical trial over the duration of clinical trial.

① In case the patient or the patient's legally authorized representative agrees to withdraw the consent to participation in this clinical trial
② In case the patient is found to have violated the inclusion/exclusion criteria
③ In case the patient is found to have violated the important study plan specified in the clinical trial protocol
④ In case the continuous administration and observations are not appropriate for this trial due to AE or SAE according to the investigator's judgment
⑤ In case the investigator has difficulty in administering the investigational product to the patient
⑥ In case the investigator judges that follow-up of the patient is impossible
⑦ In case the patient took the prohibited concomitant medication; or in case the investigator judges that the patient needs treatment with the prohibited concomitant medication
⑧ In case the investigator decides that it is not appropriate for the patient to continue to participate in this trial

14.2. Treatment After the Termination of Clinical Trial

The long-term safety and efficacy of GV1001 have not been established. Therefore patients should not be additionally administered GV1001 after the termination of this clinical trial.

The medical treatment for patients who have finished this clinical trial are patient to the medical treatment standards for general dementia patients. Patients who dropped out of this clinical trial or failed to have satisfactory effectiveness even after the administration of the investigational product should be guided to receive another appropriate treatment. Patients who have completed this clinical trial should be advised to receive medical care under the instructions of their physician at any time in preparation for unexpected delayed adverse events.

15. STATISTICAL ANALYSIS METHOD

15.1. Statistical Analysis Plan

15.1.1. Definition of Analysis Set

Analysis set is defined as follows:

1) Safety analysis set: the patients who have been randomized and undergone safety evaluation once or more among those who have been administered the investigational product at least once.

2) FAS (Full Analysis Set): the patients whose data of primary efficacy variable can be obtained among those who have been administered the investigational product at least once.

3) PPS (Per-Protocol Set): the patients who have completed this trial without violation of the protocol among those included in FAS group.

15.1.2. General principle of statistics

All statistical significance tests will be conducted in the form of 2-tailed tests at the 5% significance level (α).

The major analysis of efficacy data obtained from the patients participating in this clinical trial will be carried out in the FAS in principle.
The analysis of safety data is carried out on the Safety analysis set.

15.1.3. Analysis of Demographic Data and Baseline Features Data

In order to test statistical difference in demographic and health conditions between treatment group and control group at each dose, the mean, standard deviation, minimum value, and maximum value will be obtained for continuous data and they will be analyzed between groups using ANOVA. For categorical data, frequency will be obtained between groups, and they will be analyzed using Chi-square test, Fisher's Exact test, etc.
In order to compare pure treatment effect between treatment group and control group at each dose, whether there are differences in baseline values including demographics between groups will be examined.

15.1.4. Efficacy analysis

15.1.4.1. Primary efficacy analysis

1) Change from baseline in SIB score at Visit 15 (Week 24)

For SIB Change from baseline in SIB at Visit 15 (Week 24) compared to the baseline between treatment group and control group at each dose, the mean, standard deviation, minimum value, and maximum value will be presented; and whether there are differences in variation at the baseline and at the end point between treatment group and control group at each dose will be examined through the t-test or the Wilcoxon's rank sum test according to the layered testing strategy.

15.1.4.2. Secondary efficacy analysis

For K-MMSE, CDR-SOB, NPI, GDS, and ADCS-ADL-severe variations at the time point of 24 weeks compared to the baseline between treatment group and control group at each dose, the mean, standard deviation, minimum value, and maximum value will be presented; and whether there are differences in variation at the baseline and at the time point of 24 weeks between treatment group and control group at each dose will be examined through the t-test or the Wilcoxon's rank sum test according to the layered testing strategy. CIBIC-plus will be examined though the chi-square test.

1) Change from baseline in Korean Mini-Mental State Examination (K-MMSE) score at Visit 15 (Week 24)
2) Change from baseline in Clinical Dementia Rating-Sum of Box (CDR-SOB) score at Visit 15 (Week 24)
3) Change from baseline in Neuropsychiatric Inventory (NPI) score at Visit 15 (Week 24)
4) Change from baseline in Global Deterioration Scale (GDS) score at Visit 15 (Week 24)
5) Change from baseline in Alzheimer's Disease Cooperative Study-Activities of Daily Living-severe (ADCS-ADL-severe) score at Visit 15 (Week 24)

6) Change from baseline in Clinician Interview-Based Impression of Change-Plus (CIBIC-plus) score at Visit 15 (Week 24)

15.1.5. Safety analysis

15.1.5.1. AEs

AEs are defined as all harmful and unintended signs (e.g.: abnormal laboratory values), symptoms or diseases which occur in patients who are treated with the investigational product. It is not that AEs should always have a causal relationship with the investigational product. However, changes that originate from the progress or occurrence of normal disease are not considered AEs if the frequency and seriousness is not seriously different from the expected level. Whether there are differences in the incidence rate of AEs between groups will be analyzed using the Pearson's chi-square test or the Fisher's exact test. In addition, frequency, percentage, and number of incidence regarding AEs will be presented by physical organ and recommended term for each group.

15.1.5.2. Laboratory tests

With regard to whether each laboratory test result has any change after the termination of the investigational product use versus before treatment (screening), the descriptive statistics of the results observed will be presented per administration group. At the termination of the investigational product use versus before treatment (screening), in addition, whether there is any difference in variation between groups will be analyzed. Regarding laboratory tests, intragroup and intergroup comparisons will also be carried out.

- Intragroup comparison: For continuous variables, the descriptive statistics describing baseline, value at end time, and difference between baseline and end time will be presented; and the mean variation will be analyzed using the paired t-test or the Wilcoxon's signed rank test. For categorical variables, a contingency table will be completed, and it will be analyzed using the McNemar's test.

- Intergroup comparison: For continuous variables, difference between baseline and end time will be compared and analyzed using the ANOVA or the Kruskal-Wallis test. For categorical variables, homogeneity between treatment groups will be analyzed using the Pearson's chi-square test, the Fisher's exact test, etc.

With regard to each laboratory test result, in addition, the frequency and ratios of patients, who turn normal/ clinically non-significant abnormality into clinically significant abnormality from

before treatment (screening) to the termination of the investigational product use, will be presented for each treatment group.

15.1.5.3. Vital signs

For vital signs, the descriptive statistics will be presented for each time point of visit. With regard to whether there is any change after the termination of the investigational product use compared to before treatment (screening), the descriptive statistics of the results observed will be presented.

At the termination of the investigational product use compared to before treatment (baseline), in addition, whether there is any difference in variation between groups will be analyzed. Regarding vital signs, intragroup and intergroup comparisons will also be carried out.

- Intragroup comparisons: For continuous variables, the descriptive statistics describing baseline, value at end time, and difference between baseline and end time will be presented; and the mean variation will be analyzed using the paired t-test or the Wilcoxon's signed rank test. For categorical variables, a contingency table will be completed, and it will be analyzed using the McNemar's test.

- Intergroup comparisons: For continuous variables, difference between baseline and end time will be compared and analyzed using the ANOVA or the Kruskal-Wallis test. For categorical variables, homogeneity between treatment groups will be analyzed using the Pearson's chi-square test, the Fisher's exact test, etc.

15.1.5.4. Physical examination

For physical examinations, comparisons will be made between the physical examination results measured after the termination of the investigational product use versus before treatment (baseline), and then this will be summarized in frequency and percentage for each administration group to identify normality (including NCS)/ CS changes.

15.1.6. Handling of dropouts or missing values

If a patient who drops out of this clinical trial appears, the cause and date of dropout should be recorded in the CRF and the medical chart. If a missing value of data occurs during FAS analysis or a patient drops out of the clinical trial before its termination, the most recently

obtained data will be analyzed as if it was obtained at that relevant time (Last Observation Carried Forward Method).
However, LOCF will not be applied for PPS analysis. In case of safety evaluation items, the missing values will not be replaced.

15.1.7. Handling of protocol violations

The principal investigator and the subinvestigator should be well-acquainted with the protocol and thoroughly carry out the clinical trial procedures as specified in the protocol so that any protocol violation does not occur. In order to comply with the schedules for the administration of the investigational product and tests in this clinical trial, the investigator should take appropriate measures, such as written notification or telephone monitoring related to visit time, so that the patient can attend the visit on the date. On the other hand, the inevitable protocol violation should be handled as follows:

In case of a serious violation of the clinical trial protocol, the patient should be eliminated from the analysis (excluding PPS). Refer to the items below:

① In case the patient has no signed consent form.
② In case the patient is found to have violated the inclusion/exclusion criteria
③ In case the patient took the prohibited concomitant medication over the duration of clinical trial
④ In case the primary efficacy evaluation tests at the time of the initiation-termination of this trial were missing.

When it comes to minor protocol violations, which are judged to have no effect on the interpretation of the clinical trial results, the degrees and causes of violations or delays should be exactly recorded. Whether this affected this clinical trial will be comprehensively considered through the blind meeting prior to Database Lock, and will be included in the PPS analysis.

15.1.8. Scheduled interim analysis and data monitoring

The establishment of the Interim Analysis and Data Monitoring Committee was not planned in this trial. However, if a series of or a lot of unexpected serious side effects are reported, or the "unblinding" and early analysis of its results after discontinuation of this clinical trial are judged to be helpful or reduce risks for the patient, an independent data monitoring committee consisting of 3 persons or more not related to this clinical trial can be organized and operated. The establishment and organization of the committee should be implemented in accordance

with separate rules, and the committee should exclude the clinical investigator and analyze all data up to the time point of discontinuation.

16. SAFETY EVALUATION CRITERIA, EVALUATION METHODS AND REPORT METHODS INCLUDING ADVERSE EVENTS

16.1. Definition of Adverse Events

1) Adverse Event (AE)

AEs are defined as all harmful and unintended signs (e.g.: abnormal laboratory values), symptoms or diseases which occur in patients who are treated with the investigational product. It is not that AEs always should have causal relationship with the investigational product. AEs include but are not limited to the following items:

• Clinically significant abnormal test results
• Clinically significant symptoms or signs
• Changes in physical examination results
• Hypersensitivity
• Progress/aggravation of existing diseases.

2) Adverse Drug Reaction (ADR)

ADRs refer to all harmful and unintended reactions that are caused by a dose of the investigational product, whose causal relationship with the investigational product cannot be excluded.

3) Unexpected ADR

Unexpected ADRs mean deviation from the aspect of ADRs or the degree of their risks in view of available medication-related information such as Investigator's Brochure or Drug Labeling.

4) Serious AE/ADR

Serious AEs/ADRs refer to any of the following AEs or ADRs caused by a dose of the investigational product:

① In case of death or life-threatening
② In case the patient needs inpatient hospitalization or prolongation of existing hospitalization
③ In case the AEs/ADRs result in persistent or significant disability or incapacity
④ In case the fetus has congenital anomaly or birth defect

⑤ In case there are cases where other medically important situations such as drug dependence or abuse, or blood diseases, occur, in addition to the cases of ① to ④ above

In case there is a situation that is judged to have a medically significant effect on the patient's safety and health, even if it does not refer to the situations listed above, whether it is regarded as a SAE is determined under the medical judgment of the physician in charge and relevant experts, and then appropriate action should be taken accordingly.

In this clinical trial, hospitalization planned prior to participation in this clinical trial, hospitalization for beauty or convalescence purposes, hospitalization for usual treatment, etc. are not regarded as SAEs. The visit to an emergency room that lasts more than 24 hours is considered a SAE, but even if the visit to an emergency room is less 24 hours, it can be regarded as a SAE at the discretion of the investigator.

16.2. Collection and Recording of Adverse Events

- After randomization, AEs should be collected over the duration of this clinical trial from the time points since the administration of the investigational product to the final visit.
- AEs should be reported including the names of AEs, duration (start date and date of disappearance), severity, causal relationship with the investigational product, related measures, results, remedial treatment, and whether to have SAEs.

- When recording AEs, the investigator should record a comprehensive diagnostic name or symptom using standard medical terms rather than recording each symptom or sign.

- When it comes to AEs occurring during this clinical trial, a follow-up should be carried out until they disappear, show a stable outcome, or are lost to follow up.

16.3. Evaluation of Adverse Events

16.3.1. Severity evaluation

The severity of each AE/ADR is evaluated according to the following basis:

Grade 1 (Mild) : The patient can endure it easily.
Grade 2 (Moderate) : Considerably hinders the patient's daily living activities
Grade 3 (Severe) : Impossible for the patient to engage in normal activities of daily living.

The definition of activities of daily living (ADL) is as follows:
- All activities in daily life to manage one's body
(e.g.: taking a bath, putting on and taking off clothes, eating, taking medicines, sanitation, self-help abilities)

AEs or SAEs can be all evaluated as severe according to the definition specified above, but severe adverse events do not necessarily mean serious adverse events.

16.3.2. Evaluation of causal relationship

The investigator should determine whether incidence of an AE is related with the administration of the investigational product according to the following criteria: 1) to 5) of the following criteria (Definitely related, Probably related, Possibly related, Unlikely/remote, Unassessable) are considered "related" between the administration of the investigational product and AEs; and only 6) (Definitely not related/none) is judged as "not related" between the administration of the investigational product and AEs.

１) Definitely related

- If there is evidence that the investigational product has been used
- If the time sequence between the administration of the investigational product and onset of an AE is appropriate
- If an AE is explained most plausibly by the administration of the investigational product among any other reasons
- If an AE disappears after the administration of the investigational product is discontinued
- If the outcome of rechallenge (only if possible) turns out to be positive
- If an AE shows a pattern which is consistent with the information already known about the investigational product or other medications of the same class

２) Probably related

- If there is evidence that the investigational product has been used
- If the time sequence between the administration of the investigational product and onset of an AE is appropriate
- If an AE is explained more plausibly by the administration of the investigational product than any other reason

- If an AE disappears after the administration of the investigational product is discontinued

3) Possibly related

- If there is evidence that the investigational product has been used
- If the time sequence between the administration of the investigational product and onset of an AE is appropriate
- If it is judged that an AE is caused by the investigational product use in the same level as other potential reasons
- If an AE disappears in case the administration of the investigational product is discontinued

4) Unlikely/remote

- If there is evidence that the investigational product has been used
- If there is another more probable reason for an AE
- If the outcome of discontinuation of the investigational product use (if carried out) turns out to be negative or vague
- If the outcome of rechallenge (if carried out) turns out to be negative or vague

5) Unassessable

- If there is insufficient evidence to determine relevance
- If the quality of the evidence is low or the data is inconsistent

6) Definitely not related/none

- If the patient has not been treated with the investigational product
- If the time sequence between the administration of the investigational product and onset of an AE is not appropriate
- If there is another definite reason for an AE

16.3.3. Determination of whether an AE is unexpected

The investigator and GemVax & KAEL Co., Ltd. should determine whether an AE is expected or unexpected as long as it is judged as related to the investigational product.

- Expected: For the investigational product, the AEs with the nature, severity, frequency and characteristics which are consistent with them of the AEs described in the current Investigator's Brochure or the current clinical trial protocol are designated as "Expected."
  → For commercial drugs, the AEs specified in the current pharmaceutical drug's product manual or the applicable country' labeling are designated as "Expected."

- Unexpected: AEs not belonging to the scope of "Expected"

16.4. Reporting of Serious Adverse Events

The principal investigator and subinvestigator should report to the sponsor about all SAEs occurring over the duration of clinical trial via telephone, FAX or e-mail within 24 hours of recognition, irrespective of whether or not they are related to the investigational product used in this clinical trial, and should report to the Institutional Review Board (IRB) in accordance with the regulations of the relevant institution. When they are first reported, all the items in the SAE report form should be included and this should be attached, if necessary, in a separate form prescribed by the IRB when notifying it. In addition, they should also be recorded on the page of AEs in the electronic CRF (e-CRF).

In case a suspected unexpected serious adverse drug reaction (SUSAR) occurs, the sponsor (or his/her designee) should notify the Minister of Food and Drug Safety in accordance with the Korean Good Clinical Practice (KGCP) until the deadline according to the following division upon being reported from the subinvestigator. If the SUSAR causes death or threatens one's life, the sponsor (or his/her designee) should report this fact within 7 days from the day when being reported or coming to know it; and should additionally report detailed information within 8 days of the initial report date. In case of other all serious and unexpected ADRs, the sponsor (or his/her designee) should report this fact within 15 days from the day when being reported or coming to know it. The sponsor should submit the ADR report of Appendix no. 77 with the attached ADR summary such as the CIOMS-I form as well as the attached SAE report form separately completed by the investigator to the Minister of Food and Drug Safety. If there is an additional information, it should be reported until the termination of the relevant ADR (the disappearance of the relevant ADR or the follow-up no longer possible).

<u>Non-serious AEs</u>

Non-serious AEs should be recorded on the page of AEs in the e-CRF. The principal investigator should collect and review all non-serious AEs occurring over the duration of this clinical trial after the completion of the trial, and should evaluate them in the clinical trial result report.

AEs occurring after the termination of treatment

An AE considered to be due to the investigational product used in this clinical trial, even if it occurs after the termination of treatment, should be reported in accordance with the procedures above.

Contact a person in charge of safety information at GemVax & KAEL Co., Ltd. (Tel: +82-70-4738-9659, FAX: +82-70-4758-0370, E-mail: safety@kaelgemvax.com)

(1) Obligation of principal investigator

The principal investigator should immediately report to the IRB or the sponsor upon a serious ADR incurred during the clinical trial.

(2) Obligation of subinvestigator

The subinvestigator should immediately report to the principal investigator or the sponsor upon a serious ADR, etc. incurred during the clinical trial.

(3) Obligation of IRB

In case unexpected serious risks such as serious ADRs, etc. occur in patients, the IRB can determine the early termination or the temporary discontinuation of this clinical trial. In this case, the IRB should notify the director of study center or the principal investigator of the decision and the cause.

(4) Obligation of sponsor

In case a serious and unexpected ADR is reported by the principal investigator or the subinvestigator, the sponsor should submit the ADR report with an attached copy of the report submitted by the principal investigator or the subinvestigator to the Minister of Food and Drug

Safety within the prescribed deadline. If multiple study centers conduct this clinical trial, the sponsor should also immediately notify the relevant study centers.

16.5. Pregnancy

In this clinical trial, the patient who is found to become pregnant during the treatment period should discontinue the administration of the investigational product, which leads to the end of treatment (EOT). If the pregnancy is found within up to 90 days after the treatment period and the EOT, there should be a separate report until the end of the trial. However, follow-up should still be carried out even in case of the EOT due to pregnancy.

17. OTHER MATTERS TO CONDUCT THIS TRIAL IN A SAFE AND SCIENTIFIC MANNER

17.1. Agreement and Compliance with the Clinical Trial Protocol

The investigator should comply with the clinical trial protocol when conducting the clinical trial. The clinical trial should not be conducted differently from the clinical trial protocol, except for the case where the immediate elimination of risk factors is required for patients. If protocol violations occur, the details and cause should be recorded. Even if the investigator determines that a violation of the protocol may improve the performance of the clinical trial, any amendment should not be applied before there is the agreement with the sponsor and the approval of the IRB (including the Minister of Food and Drug Safety if necessary) about the amendment.

17.2. Approval and Amendment of Clinical Trial Protocol

For the protocol to be approved or amended, an approval of the protocol or the amended protocol should be obtained by phase from the IRB. An approval from the Minister of Food and Drug Safety may be also required if necessary. Patients should be involved in this clinical trial before the initial approval of the protocol.

17.3. Consent Procedure of Patients

The patient information sheet and the patient consent form (ICF) can be used after the approval from the IRB. The investigator should obtain consent from patients in accordance with the ethical principle based on the Declaration of Helsinki and the KGCP. Before performing all clinical trial procedures, the investigator should fully inform ptients (or their representatives) of this clinical trial and gain written consent from them. The investigator should keep the originality of signed ICF into the investigator file, and the copy of the signed ICF and the informed ptient information sheet should be offered to each ptient (or the patient's

regally authorized representative). The process of subeconsent should be recorded in the source document.

If it is impossible for the patient to give consent, the consent should be obtained from the patient's legally authorized representative. If both the patient and the patient's legally authorized representative are unable to read writing, an observer should participate in the entire process of consent. After the patient and the representative agree to participate in this clinical trial verbally and sign the ICF if possible, the observer should sign the ICF to confirm that the information specified in the ICF has been exactly explained and understood to the patient and the representative.

If there is no legally authorized representative, the role should be assumed in the order of a spouse, a lineal ascendant, and a direct descendant. If there are several lineal ascendants or direct descendants, consultation is needed, and if no agreement is reached, the oldest should become a legally acceptable representative.*)
*Paragraph 2, Article 16 of the Act on Bioethics and Safety.

If the patient information sheet and the patient consent form are amended, the investigator should obtain approval from the IRB and consent from patients (or their representatives) again. At this time, the person to be notified, and the date and contents of notification should be recorded in the source document.
Authorized

17.4. Measures for Protection of Patient's Safety

The investigator should take the rights and welfare of patients based on the Declaration of Helsinki into account when conducting this clinical trial; and the patients participating in this clinical trial should be fully informed of the KGCP, the clinical trial protocol, etc. before the start of this clinical trial. In order to thoroughly evaluate patient eligibility and incidence of AEs, the investigator should have enough time to carry out interview and tests for each patient.

The principal investigator should periodically report AEs, the progress, status, and results of this clinical trial to the sponsor, and the sponsor should periodically manage the progress of this clinical trial.

17.5. Medical Treatment and Medical Treatment Standards for Patients After Clinical Trial

The investigator should ensure that the patients who dropped out of or showed no response in this clinical trial can receive another appropriate treatment, and should also guide even the patients who have completed this clinical trial to be patient to an appropriate alternation treatment if continuous treatment is judged as necessary.

17.6. Patient Compensation Regulation

If a damage is caused in the process of the corrective action for AEs incurred due to the investigational product use or developed AEs, the sponsor will compensate the patient for the damage directly caused by the investigational product in accordance with the patient compensation regulation.

17.7. Clinical Trial Documents and Preservation of Documents

17.7.1. CRF and source documents

Clinical trial documents will be collected using e-CRF. The source documents-based data on the e-CRF should be consistent with source documents. The investigator should ensure that all documents entered in the e-CRF are accurate, complete, readable and timely.

The monitor will compare e-CRF and source documents; and if there is any difference, the monitor will inform the investigator of this and ask for an appropriate modification. Only the investigator or his/her designee is entitled to enter or modify e-CRF and source documents.

17.7.2. Accessibility to source documents

The sponsor, monitor and auditor, involved in this clinical trial can read the documents of patients for the purpose of monitoring and auditing this trial and managing the progress of this trial. The investigator should know the fact that by the contract of this clinical trial has been concluded, the sponsor or the monitor or auditor from the Contract Research Organization (CRO) can read and review the patient charts and the CRFs in order to verify them. These documents should be kept so that confidentiality is ensured, and facilities and management standards for confidentiality should also be secured. The investigator should guarantee necessary support for the CRO and the sponsor.

17.7.3. Preservation of clinical trial documents

The investigator should keep all documents and records related to this clinical trial in a safe place, maintain security for them, and preserve them for 3 years from the day when the approval for investigational product registration was obtained. After the completion of the outcome report, the clinical trial documents should be transferred to the keeping manager. If

the investigator tries to discard or transfer the clinical documents, he/she should notify the sponsor in advance.

17.7.4. Audit & Inspection

In order to guarantee the compliance with the KGCP and all relevant regulations, the sponsor or his/her designee can carry out the Quality Assurance Audit for this clinical trial; and the inspections can be performed by the Ministry of Food and Drug Safety. The investigator should receive appropriate notice before having to respond to audits and inspections, and should allow the auditor or the inspector to directly access all documents related to this clinical trial and agree to have time to discuss findings and all relevant issues.

17.8. Confidentiality of Clinical Trial Documents and Patient Records

All clinical trial results and documents should be kept secret. The investigator, the CRO, and the sponsor's person in charge should not expose all or any information related to this clinical trial without the signed approval of the sponsor.

All or any records in which patients can be identified should be kept confidential. All documents related to this clinical trial, including e-CRF, should be recorded and classified as the patient identification code instead of the patient name. If the clinical trial results are published, the identity of patients will remain confidential.

17.9. Monitoring of Study Center

Monitoring should be carried out in order to verify that the rights and well-being of human patients are protected, and the reported trial data are accurate, complete, and verifiable in comparison with source documents, and also the conduct of the study should be in compliance with the currently approved protocol and with the Article 30 and Annex 4 Regulations on Korea Good Clinical Practice in the Regulation on Safety of Pharmaceuticals, etc.

Trial monitoring will be conducted through periodic site visits and via telephone calls by the monitors from the CRO and GemVax & KAEL Co., Ltd, in order to assess the progress of this clinical trial and check if the investigator has achieved his/her duty according to the clinical trial protocol and regulations. In case of site visits, the monitors should basically check the originality of patient information, e-CRFs, drug records, data archives, etc.; and if there are any inconsistencies in the clinical trial records or any issues, will discuss them with the investigator.

17.10. Discontinuation of Clinical Trial

If the study center, the investigator or the sponsor's designee do not comply with the KGCP, the clinical trial protocol, and contract details, the sponsor will promptly rectify this and take an action. In case continued violations are found, the expected registration goal is not met, or the efficacy and safety information that may have a significant effect on the continuation of this clinical trial is generated, the sponsor may discontinue the relevant center's participation in this clinical trial.

17.11. Reporting and Publishing of Clinical Trial Results

When documents from all study centers have been completely analyzed, the sponsor should prepare a report and then inform the investigators of the clinical trial results.

All data and results obtained over the duration of this clinical trial should be owned by the sponsor, and the sponsor is entitled to announce the trial results at any time. The investigators should not publish, announce or release any information related to the trial results without the written prior consent of the sponsor, and should ensure that the subinvestigators can comply with this as well. In order to use only accurate and verified materials, the investigators should provide the sponsor with all drafts or manuscripts completed before publication or presentation and discuss them with the sponsor, and should postpone the presentation until the written approval is obtained.

As for a multi-center clinical trial, the investigators should agree that their center or some other centers do not announce trial results before the results collected from all study centers are announced. However, exceptions are made in the cases officially recognized by the principal investigators of all study centers and the sponsor.

18. REFERENCES

1) Albert MS, DeKosky ST, Dickson D, Dubois B, Feldman HH, Fox NC, et al. The diagnosisof mild cognitive impairment due to Alzheimer's disease: recommendations from the NationalInstitute on Aging-Alzheimer's Association workgroups on diagnostic guidelines forAlzheimer's disease. Alzheimers Dement. 2011;7:270-279.
2) McKhann GM, Knopman DS, Chertkow H, Hyman BT, Jack CR Jr, Kawas CH, et al. Thediagnosis of dementia due to Alzheimer's disease: recommendations from the NationalInstitute on Aging-Alzheimer's Association workgroups on diagnostic guidelines forAlzheimer's disease. Alzheimers Dement. 2011;7(3):263-269.

3) Hughes CP, Berg L, Danziger WL, Coben LA, Martin RL. A new clinical scale for thestaging of dementia. Br J Psychiatry. 1982;140:566-572.
4) Morris JC. The Clinical Dementia Rating (CDR): Current version and scoring rules.Neurology. 1993;43(11):2412-2414. Available at:
5) http://www.biostat.wustl.edu/~adrc/cdrpgm/
6) Rosen WG, Mohs RC, Davis KL. A new rating scale for Alzheimer’s disease. Am JPsychiatry. 1984;141:1356-1364.
7) Mohs RC, Knopman D, Petersen RC, Ferris SH, Ernesto C, Grundman M, et al. Developmentof cognitive instruments for use in clinical trials of antidementia drugs: additions to theAlzheimer’s Disease Assessment Scale that broaden its scope. The Alzheimer’s DiseaseCooperative Study. Alzheimer Dis Assoc Disord. 1997;11(Suppl 2):S13-S21.
8) Galasko D, Bennett D, Sano M, Ernesto C, Thomas R, Grundman M, et al. An inventory toassess activities of daily living for clinical trials in Alzheimer’s disease. The Alzheimer’sDisease Cooperative Study. Alzheimer Dis Assoc Disord. 1997;11(Suppl 2):S33-S39.
9) Galasko D, Kershaw PR, Schneider L, Zhu Y, Tariot PN. Galantamine maintains ability toperform activities of daily living in patients with Alzheimer's disease. J Am Geriatr Soc.2004;52(7):1070-1076.
10) Pfeffer RI, Kurosaki TT, Harrah CH, Chance JM, Filos S. Measurement of functionalactivities in older adults in the community. J Gerontol. 1982;37(3):323-329.
11) Cummings JL. The Neuropsychiatric Inventory: assessing psychopathology in dementiapatients. Neurology. 1997;48(5 Suppl 6):S10-S16.
12) Cummings JL, Mega M, Gray K, Rosenberg-Thompson S, Carusi DA, Gornbein J. TheNeuropsychiatric Inventory: comprehensive assessment of psychopathology in dementia.Neurology. 1994;44(12):2308-2314.
13) Lucey BP, Bateman RJ. Amyloid- β diurnal pattern: possible role of sleep in Alzheimer’sdisease pathogenesis. Neurobiol Aging. 2014; May15. Pii:S0197-4580(14)00350-9.Doi:10.1016/j.neurobiolaging.2014.03.035. [Epub ahead of print].
14) Vanderstichele H, Bibl M, Engelborghs S, Le Bastard N, Lewczuk P, Molinuevo JL, et al.Standardization of preanalytical aspects of cerebrospinal fluid biomarker testing forAlzheimer's disease diagnosis: A consensus paper from the Alzheimer's BiomarkersStandardization Initiative. Alzheimers Dement. 2012;8(1):65-73.
15) Bernhardt, S. L., Gjertsen, M. K., Trachsel, S., Moller, M., Eriksen, J. A., Meo, M., Buanes, T., and Gaudernack, G. (2006) Telomerase peptide vaccination of patients with non-

resectable pancreatic cancer: A dose escalating phase I/II study. British journal of cancer 95, 1474-1482

16) Yang Yeong-sun, Yang Hyeon-deok, Hong Yun-jeong, Kim Jeong-eun, Park Mun-ho, Na Hae-ri, Han Il-woo, Kim Sang-yun. Activities of Daily Living and Dementia. Dementia and Nerocognitive Disorders. 2012;11:29-37
17) Lee Jeong-jae, Lee Seok-beom. Recent Advances in Diagnosis and Treatment of Alzheimer's Disease. Korean J Biol Psychiatry. 2016;23(2):48-56
18) Han Su-jeon, Baek Nam-jong. Drug Therapy for Dementia. Brain & Neuro Rehabilitation. 2015; 8: 19-23
19) Gu Bon-dae, Kim Sin-gyeom, Lee Jun-yeong, Park Gi-hyeong, Shin Jun-hyeon, Kim Gwang-gi, Yun Yeong-cheol, Lee Yeong-min, Hong Chang-hyeong, Seo Sang-won, Na Deok-ryeol, Kim Seong-yun, Jeong Hae-gwan, Kim Do-gwan, Lee Jae-hong, Kim Sang-yun, Yeon Byeon-gil, Kim Su-yeong, Han Seol-hee. Clinical practice guideline for dementia by Clinical Research Center for Dementia of South Korea. J Korean Med Assoc 2011 August; 54(8): 861-875
20) Choi Seong-hye, Na Deok-ryeol, Lee Byeong-hwa, Ham Dong-seok, Jeong Ji-hyang, Jeong Yong, Gu Eun-jeong, Ha Chung-geon, Ahn Seong-shin. Validity of Korean-Global Deterioration Scale. Journal of the Korean Neurological Association. 2002;20(6):612-617
21) The Validity and Reliability of the Korean version of Severe Impairment Battery. HR Na, JW Lee, SB Ko, SM Park, SH Lee, DW Yang, IW Han, DH Kim, MJ Baek, M.S., JS Lee, JS Kim, M.S, SY Kim. Dementia and Neurocognitive Disorders 2006; 5:70-6
22) Korean Association for Geriatric Psychiatry. Korean-Dementia Rating Scale.
23) Korean Dementia Association. A Clinical Approach for Patients with Dementia.

19. ANNEX LIST

ANNEX 1. Study Centers and Investigators
ANNEX 2. Patient Information Sheet and Patient Consent Form
ANNEX 3. Patient Compensation Regulation
ANNEX 4. NINCDS-ADRDA
ANNEX 5. DSM-IV
ANNEX 6. SIB
ANNEX 7. K-MMSE
ANNEX 8. CDR-SOB
ANNEX 9. CIBIC Plus
ANNEX 10. NPI
ANNEX 11. GDS

ANNEX 12. ADCS-ADL-severe

ANNEX 13. Declaration of Helsinki
