# Supplementary material for: Efficacy and safety of GV1001 in patients with moderate-to-severe Alzheimer’s disease already receiving donepezil: a phase 2 randomized, double-blind, placebo-controlled, multicenter clinical trial
Source: Alzheimers Res Ther. 2021 Mar 26;13:66. doi: 10.1186/s13195-021-00803-w (PMC7995588; doi:10.1186/s13195-021-00803-w)
Supplement: Supplementary file 2 — Additional file 2: Table S1. Inclusion and exclusion criteria of this clinical trial. Table S2. Participant number of each group. Table S3. Observed mean total score of each outcome. Table S4. Summary of CIBIS and CIBIC-plus distributions in full analysis set. Table S5. Least mean difference from placebo in full analysis set. Table S6. Least mean difference from placebo in per-protocol population. Table S7. Overall summary of treatment-emergent adverse events: safety set population. Table S8. Most frequent treatment-emergent adverse events that occurred in > 2 patient overall: safety set population. Figure S1. GV1001 (1 mg/kg) or an equivalent volume of 0.9% saline was subcutaneously injected into old 3xTg-AD mice (B6:129-Psen1tm1Mpm Tg[APPSwe, tauP301L]1Lfa/Mmjax) from the age of 21 months until the mice were deemed ready for sacrifice according to the CCAC guidelines on selecting an appropriate endpoint in experiments using animals for research, teaching, and testing. The injections were administered three times a week until the endpoint. The neurobehavioral functions were evaluated every 3 days until the endpoint using the Y-maze test and passive avoidance task. A. In the Y-maze test, which was used to measure the willingness of rodents to explore new environments and therefore quantify the cognitive deficits,1,2 compared with 0.9% saline, 1 mg/kg GV1001 significantly improved the percentage of spontaneous alternations among the Y-maze arms. B and C. In the passive avoidance tasks, which is a fear-aggravated test used to evaluate learning and memory, the mice received an electric shock when they entered the dark compartment3,4; therefore, the latency to enter the dark compartment and number of errors reflected the learning and memory of the mice. The latency to enter the dark compartment and number of errors were significantly improved following treatment with 1 mg/kg GV1001 (B and C, respectively). D. To confirm that GV1001 can enter the brain, 1 mg/kg of GV1001 [file 13195_2021_803_MOESM2_ESM.docx]

**Additional file 2**

**Table S1. Inclusion and exclusion criteria of this clinical trial**

**Table S2. Participant number of each group**

**Table S3. Observed mean total score of each outcome**

**Table S4. Summary of CIBIS and CIBIC-plus distributions in full analysis set**

**Table S5. Least mean difference from placebo in full analysis set**

**Table S6. Least mean difference from placebo in per protocol population**

**Table S7. Overall summary of treatment-emergent adverse events: safety set population**

**Table S8. Most frequent treatment-emergent adverse events that occurred in > 2 patient overall: safety set population**

**Figure S1.**

**Table S1. Inclusion and exclusion criteria of this clinical trial**

| **Inclusion criteria**   1. 55–85 Years of age 2. Patients who satisfied diagnostic criteria for dementia in the Diagnostic and Statistical Manual of Mental Disorders, 4th edition (DSM-IV) 3. Probable Alzheimer’s disease (AD) according to National Institute of Neurological and Communicative Disorders and Stroke-Alzheimer’s Disease and Related Disorders Association (NINCDS-ADRDA) 4. Korean-Mini-Mental State Examination (K-MMSE) score ≤ 19 at screening and randomization visit 5. Global Deterioration Scale (GDS) stage 5–6 6. MRI or CT scan within 12 months prior to a screening visit that confirmed AD without any other disease that may cause dementia 7. Patients taking stable doses of donepezil for more than 3 months before the screening visit 8. Patients able to visit a hospital (as an inpatient and outpatient) and receive cognitive and other tests 9. Patients with a caregiver who can accompany them to all visits and supervise the subjects’ compliance with the procedures and study drug prescribed in the protocol and provide detailed information on the patient 10. Written informed consent from the patient or legally authorized representative |
| --- |

| **Exclusion criteria**  1) Any other cause of dementia based on MRI or CT findings and neurological examination within 12 months of screening visit  - Possible, probable, or definite vascular dementia according to the NINDS-AIREN.  - Other central nervous system diseases that may cause cognitive impairment (cerebrovascular disease including cerebrovascular dementia, Parkinsonism, Huntington's disease, subdural hematoma, normal pressure hydrocephalus, brain tumor, Creutzfeldt-Jakob disease)  - Neurological deficits such as delusions, delirium, epilepsy  2) The severity of dementia or cause of dementia thought to be due to abnormal vitamin B12, folic acid, syphilis serology, and thyroid-stimulating hormone (TSH) test results  3) Patients with current or history of significant psychiatric conditions (e.g., schizophrenia or bipolar affective disorder)  4) Patients with a history of known or suspected seizures including febrile seizures, a history of significant head trauma with loss of consciousness, or recent unconsciousness that is unexplained  5) Patients with acute or unstable cardiovascular disease, active peptic ulcer, uncontrolled hypertension, uncontrolled diabetes, insulin dependency, or any medical condition that may interfere with the completion of the clinical trial  6) Hypersensitivity to medicinal products in the study  7) History of alcohol or substance abuse or dependence (except nicotine dependence) within the last 2 years  8) Concurrent malignancies or invasive cancers diagnosed within the past 5 years except for nonmetastatic basal cell carcinoma or squamous cell carcinoma of skin, *in situ* carcinoma of the uterine cervix, or nonmetastatic prostate cancer  9) Renal impairment (creatinine clearance, CLcr < 30 mL/min)  10) Severe liver dysfunction (alanine transaminase (ALT) or aspartate transaminase (AST) > 2-fold the upper limit of normal)  11) Use of drugs other than donepezil to treat AD or other cognitive impairment  12) Use of drugs other than anticholinergic drugs, cholinergic drugs (local usage was allowed, such as pilocarpine eye drops), antidepressants (tricyclic antidepressant, MAO inhibitor) antipsychotics, or donepezil to treat AD  13) Females of childbearing age who do not consent to use medically acceptable contraception (such as surgical sterilization, intrauterine contraceptive device, condom or diaphragm, an injectable or inserted contraceptive) during the study  14) Pregnancy or breast feeding  15) Participation in other clinical trials within 4 weeks prior to this study  16) A weight of 35 kg or less  17) Patients with experience with the present study drug  18) Participation in a clinical trial for an Alzheimer dementia vaccine within the last 6 months  19) Patients considered ineligible for this study by an investigator |
| --- |

**Table S2. Participant number of each group**

|  |  | Placebo  (Group 1) | GV1001 0.56 mg  (Group 2) | GV1001 1.12 mg  (Group 3) | Total |
| --- | --- | --- | --- | --- | --- |
| Randomized set | N | 31 | 33 | 32 | 96 |
| Safety Set | N (%) | 31 (100.0) | 32 (97.0)^a^ | 32 (100.0) | 95 (99.0) |
| FAS | N (%) | 27 (87.1) | 26 (78.8) | 28 (87.5) | 81 (84.4) |
| PPS | N (%) | 26 (83.9) | 22 (66.7) | 25 (78.1) | 73 (76.0) |

^a^One participant (05-S-013) withdrew after randomization but before treatment drug administration

Abbreviation: *FAS* Full Analysis Set, *PPS* Per-Protocol Set

**Table S3. Observed mean total score of each outcome**

| Variables |  | Observed mean total score ± SD | | |
| --- | --- | --- | --- | --- |
|  |  | Placebo  (Group 1) | GV1001 0.56 mg  (Group 2) | GV1001 1.12 mg  (Group 3) |
|  |  | n=27 | n=26 | n=28 |
| SIB | Baseline | 77.8 ± 16.6 | 82.6 ± 18.0 | 78.7 ± 20.3 |
|  | Week 12 (V 9) | 76.6 ± 19.8 | 82.3 ± 20.3 | 79.4 ± 18.6 |
|  | Week 24 (V 15) | 71.5 ± 24.7 | 81.4 ± 20.9 | 78.0 ± 21.5 |
| K-MMSE | Baseline | 11.7 ± 4.7 | 14.2 ± 4.2 | 12.7 ± 4.7 |
|  | Week 12 (V 9) | 12.1 ± 5.7 | 13.3 ± 5.4 | 12.9 ± 5.8 |
|  | Week 24 (V 15) | 11.2 ± 5.5 | 13.2 ± 5.5 | 12.1 ± 6.6 |
| CDR-SOB | Baseline | 10.3 ± 4.1 | 9.3 ± 4.5 | 10.5 ± 4.1 |
|  | Week 12 (V 9) | 10.4 ± 4.4 | 9.9 ± 4.8 | 10.9 ± 4.6 |
|  | Week 24 (V 15) | 11.4 ± 5.1 | 9.6 ± 4.8 | 11.3 ± 5.1 |
| NPI | Baseline | 18.7 ± 14.5 | 14.6 ± 12.5 | 23.9 ± 16.9 |
|  | Week 12 (V 9) | 24.9 ± 17.2 | 18.3 ± 18.4 | 21.3 ± 15.7 |
|  | Week 24 (V 15) | 18.0 ± 12.6 | 18.0 ± 19.5 | 25.5 ± 22.4 |
| GDS | Baseline | 5.4 ± 0.5 | 5.2 ± 0.4 | 5.4 ± 0.5 |
|  | Week 12 (V 9) | 5.2 ± 0.6 | 5.2 ± 0.6 | 5.3 ± 0.6 |
|  | Week 24 (V 15) | 5.3 ± 0.7 | 5.2 ± 0.5 | 5.3 ± 0.6 |
| ADCS-ADL | Baseline | 33.1 ± 10.6 | 39.1 ± 11.3 | 35.9 ± 10.8 |
|  | Week 12 (V 9) | 32.0 ± 12.6 | 39.0 ± 11.9 | 35.6 ± 11.6 |
|  | Week 24 (V 15) | 29.7 ± 13.5 | 38.2 ± 12.6 | 32.3 ± 12.9 |

**Table S4. Summary of CIBIS and CIBIC-plus distributions in full analysis set**

|  | Group 1  Placebo | Group 2  (GV1001 0.56 mg) | Group 3  (GV1001 1.12 mg) | *P* value |
| --- | --- | --- | --- | --- |
| CIBIS Baseline |  |  |  | 0.6926 |
| n | 27 | 26 | 28 |  |
| 1 Normal, not at all ill | 0 (0.0) | 0 (0.0) | 0 (0.0) |  |
| 2 Borderline mentally ill | 0 (0.0) | 0 (0.0) | 0 (0.0) |  |
| 3 Mildly ill | 2 (7.4) | 1 (3.8) | 1 (3.6) |  |
| 4 Moderately ill | 10 (37.0) | 11 (42.3) | 6 (21.4) |  |
| 5 Markedly ill | 11 (40.7) | 11 (42.3) | 15 (53.6) |  |
| 6 Severely ill | 4 (14.8) | 3 (11.5) | 6 (21.4) |  |
| 7 Among the most extremely  ill patients | 0 (0.0) | 0 (0.0) | 0 (0.0) |  |
| CIBIC-plus Visit 9 (Week 12) |  |  |  | 0.4616 |
| n | 25 | 24 | 28 |  |
| 1 Very Much Improved | 0 (0.0) | 0 (0.0) | 0 (0.0) |  |
| 2 Much Improved | 0 (0.0) | 0 (0.0) | 0 (0.0) |  |
| 3 Minimally Improved | 5 (18.5) | 4 (15.4) | 5 (17.9) |  |
| 4 No change | 9 (33.3) | 15 (57.7) | 13 (46.4) |  |
| 5 Minimal worsening | 11 (40.7) | 5 (19.2) | 9 (32.1) |  |
| 6 Moderate worsening | 0 (0.0) | 0 (0.0) | 1 (3.6) |  |
| 7 Marked worsening | 0 (0.0) | 0 (0.0) | 0 (0.0) |  |
| 8 Unable to evaluate | 0 (0.0) | 0 (0.0) | 0 (0.0) |  |
| CIBIC-plus Visit 15 (Week 24) |  |  |  | 0.6545 |
| n | 27 | 26 | 28 |  |
| 1 Very Much Improved | 0 (0.0) | 0 (0.0) | 0 (0.0) |  |
| 2 Much Improved | 0 (0.0) | 0 (0.0) | 1 (3.6) |  |
| 3 Minimally Improved | 1 (3.7) | 2 (7.7) | 2 (7.1) |  |
| 4 No change | 11 (40.7) | 14 (53.8) | 14 (50.0) |  |
| 5 Minimal worsening | 13 (48.1) | 8 (30.8) | 7 (25.0) |  |
| 6 Moderate worsening | 2 (7.4) | 2 (7.7) | 4 (14.3) |  |
| 7 Marked worsening | 0 (0.0) | 0 (0.0) | 0 (0.0) |  |
| 8 Unable to evaluate | 0 (0.0) | 0 (0.0) | 0 (0.0) |  |

Abbreviation: *n* number, *CIBIS* Clinician Interview Based Impression of Severity, *CIBIC-plus* Clinician Interview-Based Impression of Change plus caregiver input

**Table S5. Least mean difference from placebo in full analysis set**

LS mean Difference from Placebo

|  |  | GV1001 (1.12 mg) vs Placebo | | |  | GV1001 (0.56 mg) vs Placebo | | |
| --- | --- | --- | --- | --- | --- | --- | --- | --- |
| Variables |  | LS mean ± SE | 95% CI | *P* value |  | LS mean ± SE | 95% CI | *P* value |
|  |  |  |  |  |  |  |  |  |
| SIB | Baseline | NA | NA | NA |  | NA | NA | NA |
|  | Week 12 (V 9) | 4.4 (2.0) | 0.4, 8.3 | 0.0303 |  | 2.9 (2.2) | -1.4, 7.3 | 0.1778 |
|  | Week 24 (V 15) | 6.6 (2.9) | 0.8, 12.4 | 0.0271 |  | 4.3 (2.5) | -0.8, 9.4 | 0.0972 |
| K-MMSE | Baseline | NA | NA | NA |  | NA | NA | NA |
|  | Week 12 (V 9) | 0.1 (0.8) | -1.5, 1.8 | 0.8582 |  | -1.0 (0.8) | -2.6, 0.6 | 0.2150 |
|  | Week 24 (V 15) | 0.3 (0.9) | -1.5, 2.0 | 0.7511 |  | -0.8 (0.8) | -2.4, 0.9 | 0.3653 |
| CDR-SOB | Baseline | NA | NA | NA |  | NA | NA | NA |
|  | Week 12 (V 9) | -0.2 (0.3) | -0.8, 0.5 | 0.5936 |  | -0.1 (0.3) | -0.8, 0.6 | 0.7716 |
|  | Week 24 (V 15) | -0.3 (0.5) | -1.3, 0.6 | 0.5181 |  | -0.6 (0.4) | -1.4, 0.3 | 0.1819 |
| NPI | Baseline | NA | NA | NA |  | NA | NA | NA |
|  | Week 12 (V 9) | -7.6 (3.1) | -13.9, -1.4 | 0.0179 |  | -4.9 (4.3) | -13.7, 3.8 | 0.2632 |
|  | Week 24 (V 15) | 1.4 (5.4) | -9.5, 12.4 | 0.7921 |  | 4.2 (5.1) | -6.1, 14.5 | 0.4185 |
| GDS | Baseline | NA | NA | NA |  | NA | NA | NA |
|  | Week 12 (V 9) | 0.0 (0.1) | -0.1, 0.2 | 0.6638 |  | 0.1 (0.1) | -0.1, 0.3 | 0.2027 |
|  | Week 24 (V 15) | 0.1 (0.1) | -0.1, 0.3 | 0.4764 |  | 0.2 (0.1) | -0.0, 0.4 | 0.0745 |
| ADCS-ADL | Baseline | NA | NA | NA |  | NA | NA | NA |
|  | Week 12 (V 9) | 1.7 (1.2) | -0.7, 4.1 | 0.1659 |  | 1.7 (1.2) | -0.6, 4.0 | 0.1478 |
|  | Week 24 (V 15) | 1.0 (1.3) | -1.5, 3.6 | 0.4199 |  | 1.1 (1.4) | -1.8, 4.0 | 0.4507 |

Abbreviation: *LS* least squre, *SE* standard error, *NA* not applicable, *SIB* Severe Impairment Battery, *K-MMSE* Korean-Mini-Mental State Examination, *CDR-SOB* Clinical Dementia Rating Scale-Sum of Boxes, *NPI* Neuropsychiatric Inventory, *GDS* Global Deterioration Scale, *ADCS-ADL* Alzheimer's Disease Cooperative Study-Activities of Daily Living

**Table S6. Least mean difference from placebo in per-protocol population**

LS mean Difference from Placebo

|  |  | GV1001 (1.12 mg) vs Placebo | | |  | GV1001 (0.56 mg) vs Placebo | | |
| --- | --- | --- | --- | --- | --- | --- | --- | --- |
| Variables |  | LS mean ± SE | 95% CI | *P* value |  | LS mean ± SE | 95% CI | *P* value |
|  |  |  |  |  |  |  |  |  |
| SIB | Baseline | NA | NA | NA |  | NA | NA | NA |
|  | Week 12 (V 9) | 4.4 (2.0) | 0.4, 8.3 | 0.0303 |  | 2.9 (2.2) | -1.4, 7.3 | 0.1778 |
|  | Week 24 (V 15) | 6.6 (2.9) | 0.8, 12.4 | 0.0271 |  | 4.3 (2.5) | -0.8, 9.4 | 0.0972 |
| K-MMSE | Baseline | NA | NA | NA |  | NA | NA | NA |
|  | Week 12 (V 9) | 0.1 (0.8) | -1.5, 1.8 | 0.8582 |  | -1.0 (0.8) | -2.6, 0.6 | 0.2150 |
|  | Week 24 (V 15) | 0.3 (0.9) | -1.5, 2.0 | 0.7511 |  | -0.8 (0.8) | -2.4, 0.9 | 0.3653 |
| CDR-SOB | Baseline | NA | NA | NA |  | NA | NA | NA |
|  | Week 12 (V 9) | -0.2 (0.3) | -0.8, 0.5 | 0.5936 |  | -0.1 (0.3) | -0.8, 0.6 | 0.7716 |
|  | Week 24 (V 15) | -0.3 (0.5) | -1.3, 0.6 | 0.5181 |  | -0.6 (0.4) | -1.4, 0.3 | 0.1819 |
| NPI | Baseline | NA | NA | NA |  | NA | NA | NA |
|  | Week 12 (V 9) | -7.6 (3.1) | -13.9, -1.4 | 0.0179 |  | -4.9 (4.3) | -13.7, 3.8 | 0.2632 |
|  | Week 24 (V 15) | 1.4 (5.4) | -9.5, 12.4 | 0.7921 |  | 4.2 (5.1) | -6.1, 14.5 | 0.4185 |
| GDS | Baseline | NA | NA | NA |  | NA | NA | NA |
|  | Week 12 (V 9) | 0.0 (0.1) | -0.1, 0.2 | 0.6638 |  | 0.1 (0.1) | -0.1, 0.3 | 0.2027 |
|  | Week 24 (V 15) | 0.1 (0.1) | -0.1, 0.3 | 0.4764 |  | 0.2 (0.1) | -0.0, 0.4 | 0.0745 |
| ADCS-ADL | Baseline | NA | NA | NA |  | NA | NA | NA |
|  | Week 12 (V 9) | 1.7 (1.2) | -0.7, 4.1 | 0.1659 |  | 1.7 (1.2) | -0.6, 4.0 | 0.1478 |
|  | Week 24 (V 15) | 1.0 (1.3) | -1.5, 3.6 | 0.4199 |  | 1.1 (1.4) | -1.8, 4.0 | 0.4507 |

Abbreviation: *LS* least squre, *SE* standard error, *NA* not applicable, *SIB* Severe Impairment Battery, *K-MMSE* Korean-Mini-Mental State Examination, *CDR-SOB* Clinical Dementia Rating Scale-Sum of Boxes, *NPI* Neuropsychiatric Inventory, *GDS* Global Deterioration Scale, *ADCS-ADL* Alzheimer's Disease Cooperative Study-Activities of Daily Living

**Table S7. Overall summary of treatment-emergent adverse events: safety set population**

| **Category** | **Placebo**  **(Group 1)**  **(n=31)** | | **GV1001 0.56 mg**  **(Group 2)**  **(n=32)** | | **GV1001 1.12 mg**  **(Group 3)**  **(n=32)** | | **Overall**  **(n=95)** | | ***P* value** |
| --- | --- | --- | --- | --- | --- | --- | --- | --- | --- |
|  | **n (%)** | **events** | **n (%)** | **events** | **n (%)** | **events** | **n (%)** | **events** |  |
| Patients with any TEAE | 16(51.6) | 38 | 18(56.3) | 34 | 15(46.9) | 44 | 49(51.6) | 116 | 0.7545^a^ |
| Patients with any ADR | 7(22.6) | 19 | 10(31.3) | 16 | 8(25.0) | 15 | 25(26.3) | 50 | 0.7213^a^ |
| Patients with any SAE | 2(6.5) | 2 | 1(3.1) | 1 | 0(0.0) | 0 | 3(3.2) | 3 | 0.3191^b^ |
| Patients with any TEAE leading to study withdrawal | 1(3.2) | 1 | 1(3.1) | 2 | 1(3.1) | 1 | 3(3.2) | 4 | 1.0000^b^ |

Abbreviations: *n* Number of patients, *TEAE* Treatment-emergent adverse event, *SAE* Severe adverse event, *ADR* Adverse drug reaction

Note: Study Group 1 = placebo (control), Study Group 2 = GV1001 0.56 mg; Study Group 3 = GV1001 1.12 mg

^a^Chi-square test; ^b^Fisher’s exact test

**Table S8. Most frequent treatment-emergent adverse events that occurred in > 2 patient overall: safety set population**

| **System Organ Class^a^**  **Preferred Term^a^** | **Placebo**  **(Group 1)**  **(n=31)** | | **GV1001 0.56 mg**  **(Group 2)**  **(n=32)** | | **GV1001 1.12 mg**  **(Group 3)**  **(n=32)** | | **Overall**  **(n=95)** | |
| --- | --- | --- | --- | --- | --- | --- | --- | --- |
|  | **n (%)** | **events** | **n (%)** | **events** | **n (%)** | **events** | **n (%)** | **events** |
| Infections and infestations |  |  |  |  |  |  |  |  |
| Nasopharyngitis | 3(9.7) | 5 | 2(6.3) | 2 | 1(3.1) | 1 | 6(6.3) | 8 |
| Cellulitis | 1(3.2) | 1 | 1(3.1) | 1 | 1(3.1) | 1 | 3(3.2) | 3 |
| Gastrointestinal disorders |  |  |  |  |  |  |  |  |
| Diarrhea | 1(3.2) | 1 | 2(6.3) | 2 | 1(3.1) | 1 | 4(4.2) | 4 |
| Abdominal pain | 1(3.2) | 2 | 1(3.1) | 1 | 1(3.1) | 1 | 3(3.2) | 4 |
| Psychiatric disorders |  |  |  |  |  |  |  |  |
| Anxiety | 1(3.2) | 1 | 2(6.3) | 2 | 1(3.1) | 1 | 4(4.2) | 4 |
| Delusion | 2(6.5) | 2 | 1(3.1) | 1 | 0(0.0) | 0 | 3(3.2) | 3 |
| Musculoskeletal and connective tissue disorders |  |  |  |  |  |  |  |  |
| Back pain | 0(0.0) | 0 | 2(6.3) | 2 | 2(6.3) | 2 | 4(4.2) | 4 |
| Arthralgia | 1(3.2) | 1 | 2(6.3) | 2 | 0(0.0) | 0 | 3(3.2) | 3 |
| Metabolism and nutrition disorders |  |  |  |  |  |  |  |  |
| Decreased appetite | 1(3.2) | 1 | 2(6.3) | 2 | 0(0.0) | 0 | 3(3.2) | 3 |
| Vascular disorders |  |  |  |  |  |  |  |  |
| Hypertension | 2(6.5) | 2 | 1(3.1) | 1 | 1(3.1) | 1 | 4(4.2) | 4 |

Abbreviations: *TEAE* Treatment-emergent adverse event, *n* Number of patients.

Note: Study Group 1 = placebo (control), Study Group 2 = GV1001 0.56 mg; Study Group 3 = GV1001 1.12 mg

^a^TEAEs are classified using MedDRA version 22.1

**Figure S1.**

**
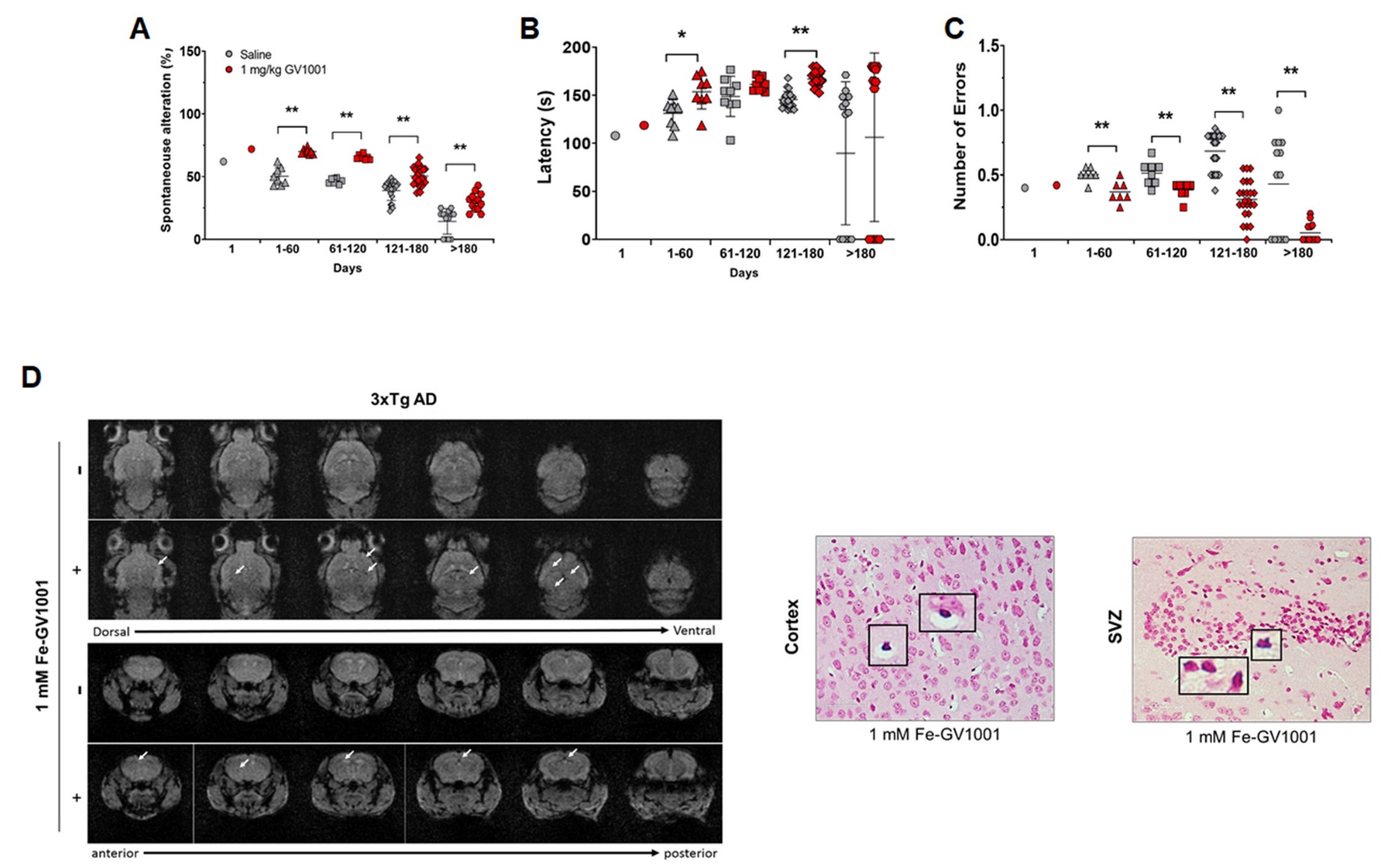
**

GV1001 (1 mg/kg) or an equivalent volume of 0.9% saline was subcutaneously injected into old 3xTg-AD mice (B6:129-Psen1tm1Mpm Tg[APPSwe, tauP301L]1Lfa/Mmjax) from the age of 21 months until the mice were deemed ready for sacrifice according to the CCAC guidelines on selecting an appropriate endpoint in experiments using animals for research, teaching, and testing. The injections were administered three times a week until the endpoint. The neurobehavioral functions were evaluated every 3 days until the endpoint using the Y-maze test and passive avoidance task. A. In the Y-maze test, which was used to measure the willingness of rodents to explore new environments and therefore quantify the cognitive deficits,^1,2^ compared with 0.9% saline, 1 mg/kg GV1001 significantly improved the percentage of spontaneous alternations among the Y-maze arms. B and C. In the passive avoidance tasks, which is a fear-aggravated test used to evaluate learning and memory, the mice received an electric shock when they entered the dark compartment^3,4^; therefore, the latency to enter the dark compartment and number of errors reflected the learning and memory of the mice. The latency to enter the dark compartment and number of errors were significantly improved following treatment with 1 mg/kg GV1001 (B and C, respectively). D. To confirm that GV1001 can enter the brain, 1 mg/kg of GV1001 conjugated with ferrocenecarboxylic acid, which we used in our previous study,^5^ was subcutaneously injected into 3xTg-AD (12-month-old) mice. It was detected as dark signals in the brain using 3T magnetic resonance imaging (white arrows) and Prussian blue staining (black boxes).

Reference

1. Kraeuter AK, Guest PC, Sarnyai Z. The Y-maze for assessment of spatial working and reference memory in mice. Methods Mol Biol. 2019;1916:105-111.
2. He Z, Guo JL, McBride JD, et al. Amyloid-beta plaques enhance Alzheimer's brain tau-seeded pathologies by facilitating neuritic plaque tau aggregation. Nat Med. 2018;24:29-38.
3. Bartus RT, Dean RL, 3rd, Beer B, Lippa AS. The cholinergic hypothesis of geriatric memory dysfunction. Science*.* 1982;217:408-414.
4. Jo S, Yarishkin O, Hwang YJ, Chun YE, Park M, Woo DH. GABA from reactive astrocytes impairs memory in mouse models of Alzheimer’s disease. Nat Med. 2014;20:886-896.
5. Park HH, Lee KY, Park DW, et al. Tracking and protection of transplanted stem cells using a ferrocenecarboxylic acid-conjugated peptide that mimics hTERT. Biomaterials. 2018;155:80-91.
